# A core outcome set for maternal and neonatal health research and surveillance of emerging and ongoing epidemic threats (MNH-EPI-COS): a modified Delphi-based international consensus

**DOI:** 10.1016/j.eclinm.2024.103025

**Published:** 2025-01-15

**Authors:** Verónica Pingray, Karen Klein, Juan Pedro Alonso, María Belizan, Magdalena Babinska, Jackeline Alger, Hellen C. Barsosio, Kara Blackburn, Olufunke Bolaji, Courtney Carson, Sofia Castiglioni, Daniele De Luca, Sangappa Dhaded, Cyril Engmann, María Fernanda Escobar Vidarte, Ramón Escuriet, Edna Kara, Caron Rahn Kim, Marian Knight, Smaragda Lamprianou, Maria Margarita Lota, Silke Mader, Lola Madrid, Alessandra L. Marcone, Agustina Mazzoni, Rangel Mirna Montenegro, Rose Mukisa-Bisoborwa, Flor M. Munoz, Uduak Okomo, Pius Okong, Vanesa Ortega, Florencia A. Salva, David A. Schwartz, Tavitiya Sudjaritruk, Laura Yates, Manal Younus, Noreen Zafar, Olufemi T. Oladapo, Mabel Berrueta, Mercedes Bonet

**Affiliations:** a Department of Maternal and Child Health, Institute for Clinical Effectiveness and Health Policy, Buenos Aires, Argentina; b Unit of Qualitative Health Research, Institute for Clinical Effectiveness and Health Policy, Buenos Aires, Argentina; c UNDP-UNFPA-UNICEF-WHO-World Bank Special Programme of Research, Development and Research Training in Human Reproduction (HRP), Department of Sexual and Reproductive Health and Research, World Health Organization, Geneva, Switzerland; d Departamento de Laboratorio Clínico, Hospital Escuela, Tegucigalpa, Honduras; e Instituto de Enfermedades Infecciosas y Parasitología Antonio Vidal, Tegucigalpa, Honduras; f Centre for Global Health Research, Maternal and Newborn Health Research, Kenya Medical Research Institute (KEMRI), Kisumu, Kenya; g Maternal, Child and Adolescent Health Program, Burnet Institute, Melbourne, Australia; h Neonatal Unit, Department of Paediatrics and African Neonatal Association, Afe Babalola University, Ado-Ekiti, Nigeria; i Pandemic Action Network, New York, United States; j NYU Grossman School of Medicine, New York, NY, USA; k Division of Paediatrics and Neonatal Critical Care, APHP-Paris Saclay University - “A. Béclère” Medical Centre, Paris, France; l Physiopathology and Therapeutic Innovation Unit-INSERME U999, Paris Saclay University, Paris, France; m Department of Neonatology, KLE Academy of Higher Education and Research, JN Medical College, Belgavi, India; n Maternal, Newborn, Child Health & Nutrition, PATH, Seattle, United States; o Departments of Paediatrics & Global Health, University of Washington Schools of Medicine & Public Health, Seattle, United States; p Unidad de Equidad Global en Salud, Departamento de Ginecología y Obstetricia, Facultad de Ciencias de la Salud, Fundación Valle del Lili, Cali, Colombia; q National Health Service, Barcelona, Spain; r National Perinatal Epidemiology Unit, Nuffield Department of Population Health, University of Oxford, Oxford, United Kingdom; s Department of Regulation and Prequalification, Pharmacovigilance Team, World Health Organization, Geneva, Switzerland; t Department of Medical Microbiology, College of Public Health, University of the Philippines, Manila, Philippines; u European Foundation for the Care of Newborn Infants (EFCNI), Munich, Germany; v Department of Infectious Disease Epidemiology and International Health, London School of Hygiene and Tropical Medicine (LSHTM), London, United Kingdom; w Observatory for Sexual and Reproductive Health (OSAR), Guatemala City, Guatemala; x White Ribbon Alliance Uganda, Kampala, Uganda; y Division of Infectious Disease, and Molecular Virology and Microbiology, Departments of Paediatrics, Baylor College of Medicine, Houston, TX, USA; z Medical Research Council (MRC) Unit, London School of Hygiene and Tropical Medicine (LSHTM), Banjul, The Gambia; aa Uganda Martyrs University (UMU), Nkozi, Uganda; ab Perinatal Pathology Consulting, Atlanta, GA, USA; ac Department of Paediatrics, Faculty of Medicine, Chiang Mai University, Chiang Mai, Thailand; ad KwaZuluNatal Research and Innovation Sequencing Platform (KRISP), University of KwaZulu-Natal, Durban, South Africa; ae Northern Genetics Service, Newcastle-upon-Tyne Hospitals NHS Foundation Trust, UK; af Iraqi Pharmacovigilance Centre, Ministry of Health, Baghdad, Iraq; ag Girls and Women's Health Initiative, Lahore, Pakistan

**Keywords:** Maternal health, Neonatal health, Epidemics, Core outcome set, Consensus

## Abstract

**Background:**

Disease outbreaks significantly affect maternal and neonatal health. Variability in reporting health outcomes hinder evidence generation. We aimed to develop a core outcome set (COS) for maternal and neonatal health research and surveillance during emerging and ongoing epidemic threats and to agree on outcomes’ definitions.

**Methods:**

We conducted a systematic review of observational and experimental studies related to epidemics to identify outcomes, and a four-stage modified-Delphi consensus. 150 international stakeholders participated in online surveys, and 24 representatives in consensus meetings. The panels were diverse, with balanced representation of professional background, gender, and geography, including civil society representatives. Outcome were included if ≥ 80% of participants scored them as critically important and ≤10% rated them as not important.

**Findings:**

The final COS includes seven main maternal outcomes—pregnancy outcome, maternal death, suspected symptomatic infection, confirmed infection, severe disease, preterm delivery, mode of birth; seven complementary maternal outcomes—antepartum haemorrhage, postpartum haemorrhage, hypertensive disorders of pregnancy, maternal sepsis, admission to intensive care unit/special units, respiratory support, depression and anxiety; 11 main neonatal outcomes—neonatal death, neonatal suspected symptomatic infection, confirmed infection, severe disease, vertical transmission, low birth weight, prematurity, congenital disorder, respiratory support, skin-to-skin contact, breastfeeding; and, four complementary neonatal outcomes—admission to neonatal intensive care unit/special units, respiratory failure, birth asphyxia, sepsis.

**Interpretation:**

This COS could contribute to standardize maternal and neonatal outcomes selection and reporting in observational and experimental studies, facilitating efficient data comparison and timely evidence-based decision-making in the context of ongoing and emerging epidemic threats.

**Funding:**

10.13039/100000865Bill & Melinda Gates Foundation (grant INV-041181) and the 10.13039/100016195UNDP/UNFPA/10.13039/100006641UNICEF/10.13039/100004423WHO/World Bank Special Programme of Research, Development and Research Training in Human Reproduction (HRP), a cosponsored programme executed by the World Health Organization (HQHRP2422779).

**Research in context**

**Evidence before this study**

The generation of scientific evidence on the effects of emerging and ongoing epidemic threats, as well as related preventive and therapeutic interventions, on pregnant women, fetuses, and neonates, is often severely delayed or entirely lacking. This leaves decision makers, such as policy makers, healthcare providers and pregnant women, without information needed to make informed care decisions, as highlighted in the COVID-19 pandemic. Establishing a set of outcomes could help standardize reporting and timely evidence generation. Before conducting this study, we searched on Pubmed (“Pregnancy” OR “Childbirth” OR “Fetal” OR “Maternofetal” OR “Perinatal” OR “Newborn” OR “Neonatal”) AND (“Outbreaks” OR “Epidemic” OR “Pandemic”) AND (“Basic outcomes” OR “Core outcomes” OR “Core outcome set” OR “Minimal set” OR “COS”), but did not identify relevant publications. A subsequent broader search using this strategy (Pregnancy [Mesh] OR Pregnancy Complications [Mesh] OR Abortion, Spontaneous [Mesh] OR Parturition [Mesh] OR Fetus [Mesh] OR DART [tiab]) AND (Epidemics [Mesh]) AND (Pregnancy Outcome [Mesh]), along with a review of all core outcome sets (COS) registered in the COMET database, also did not identified relevant publications. Although there is literature on standardizing pregnancy and newborn outcomes for immunization safety assessment, there are currently no published COS for maternal and neonatal health research specifically designed for application in the context of ongoing and emerging epidemic threats.

**Added value of this study**

We developed a COS—consisting of 18 main outcomes and 11 complementary outcomes—for evaluating maternal and neonatal health during emerging and ongoing epidemic threats across epidemiological studies, clinical studies assessing the safety and effectiveness of preventive and therapeutic interventions, and post-authorization safety surveillance. The development of this COS involved multiple stages and incorporated diverse perspectives from an international group of stakeholders including representatives from civil society, ensuring its relevance and applicability across diverse settings. This COS will contribute to minimize research waste and help facilitate timely evidence generation to enhance preparedness for future epidemics.

**Implications of all the available evidence**

This COS can help researchers in standardising outcome reporting in epidemiological studies on maternal and neonatal health, product development, and post-authorization surveillance during epidemic threats. Researchers should report this COS as a minimum, alongside any complementary and additional outcomes of interest. Once outcome sets have been defined, it is necessary to identify barriers that inhibit the implementation and adoption of the core outcome sets in various settings. Developing strategies to overcome these barriers is essential to effectively enhance the comparability and meta-analysis of study findings, thereby fostering meaningful changes in health research.

## Introduction

In recent decades, the world has experienced numerous outbreaks of infectious diseases with epidemic and pandemic potential.^1^ Pregnant and recently pregnant women, along with their babies, are frequently negatively affected by the direct and indirect effects of these outbreaks.^2^, ^3^, ^4^, ^5^, ^6^, ^7^, ^8^ As these populations require comprehensive healthcare services, the risks of maternal, perinatal and neonatal complications due to an infectious disease outbreak are exacerbated by disruptions and restrictions in access to quality care.^9^, ^10^, ^11^ In addition, outbreaks may occur with the risk to pregnant women and their babies from new pathogens being unknow, and real-time epidemiological surveillance is needed to inform the response.

Wide variations in how health outcomes are defined, measured, and reported across studies significantly limit researchers’ ability to compare, collate, and interpret findings and draw reliable conclusions.^12^, ^13^, ^14^ At the same time, pregnancy status is often not reported in health surveillance systems, and pregnant women are frequently excluded from clinical trials of new or repurposed countermeasures (i.e., vaccines and drugs).^12^^,^^13^^,^^15^^,^^16^ Consequently, the process of generating scientific evidence on how epidemic threats affect pregnant women, fetuses, and neonates, or the impact and safety of therapeutic or prophylactic interventions is typically delayed. Therefore, decision-makers, healthcare providers, pregnant women, and their families may lack the necessary evidence to make informed care decisions.^17^ This represents a substantial barrier to translating research findings into public health interventions and clinical practice.

To address the challenge of inconsistent outcome selection, a core outcome set (COS) is needed. A COS is a minimum set of outcomes recommended to be measured within a specific field of research or clinical practice.^18^ Establishing a COS for assessing maternal and neonatal health during outbreaks, epidemics, or pandemics should facilitate timely evidence generation and decision-making. Available literature reports sets of pregnancy and newborn outcomes for monitoring the safety of maternal vaccines.^19^^,^^20^ However, no standardized set of outcomes has been proposed for maternal and neonatal research or surveillance conducted during emerging and ongoing epidemics. This study aimed to develop a set of core outcomes for maternal and neonatal health research and surveillance during emerging and ongoing epidemic threats and to agree on outcomes’ definitions and measurement instruments, when applicable.

## Methods

### Study overview

The development of the MNH-EPI-COS and agreement on outcomes’ definitions were based on a systematic review to identify outcomes, and a modified-Delphi consensus process. The protocol was published and registered in Core Outcome Measures in Effectiveness Trials (COMET).^21^^,^^22^ This report follows the Core Outcome Set Standards for Reporting (COS-STAR); and an independent Technical Advisory Group provided technical guidance to this project.^23^^,^^24^

### Systematic review of maternal and neonatal outcomes

We conducted a systematic review to identify maternal and neonatal health outcomes reported in research conducted during outbreaks, epidemics, and pandemics. Experimental and observational studies, protocols, and ongoing studies published in English were included. We searched in MEDLINE, EMBASE, LILACS, SCI-EXPANDED, CINAHL, Cochrane Central Register of Controlled Trials, PsycINFO, AMED, ClinicalTrials.gov and ICTRP, between January 2015 and March 2023. Outcomes were classified into domains (Supplementary File S1).^25^ Further details on the methodology and results of this systematic review are published elsewhere.^26^^,^^27^

### Modified Delphi consensus process

**Fig. 1:**
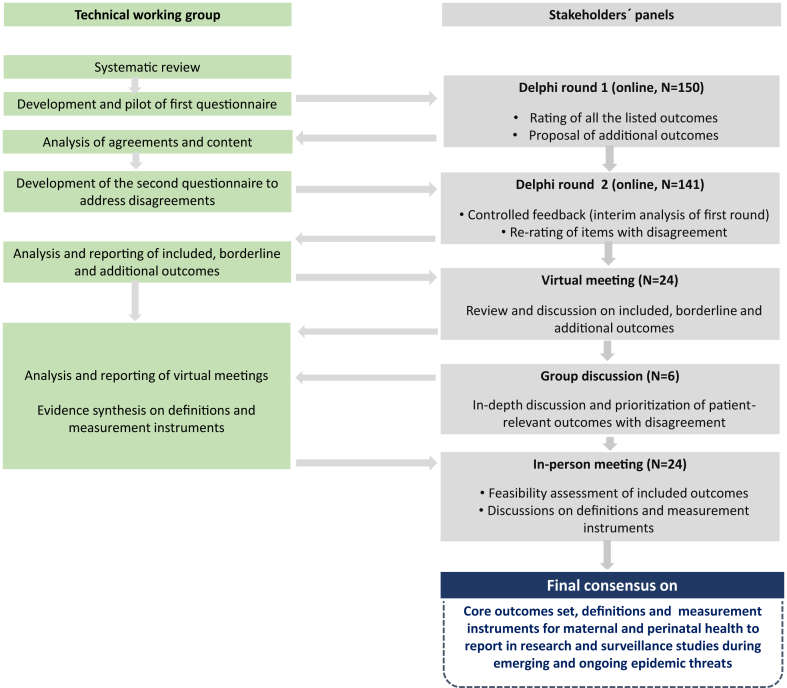
Flowchart of the Modified-Delphi study process.

Consensus was achieved through an iterative four-stage modified Delphi process (Fig. 1); which started with two rounds of online surveys; followed by two rounds of consensus meetings. The COS was developed for use in maternal and neonatal research and surveillance, particularly in studies focused on: 1) epidemiology, 2) safety and efficacy of preventive and therapeutic interventions, and 3) post-authorization surveillance, all within the context of ongoing and emerging epidemic threats.

#### Participants

A purposive sample was selected to ensure a multidisciplinary and comprehensive perspective, through diverse representation in professional backgrounds, geographical diversity, and a balanced, self-reported gender distribution. We invited 197 key stakeholders, estimating this number to account for a 20% non-response rate and a 20% attrition rate while ensuring that each panel would retain a minimum of 20 participants. This approach aligns with the RAND Corporation's guidance, which recommends approximately 18 experts with diverse expertise per panel, as well as with Boukelid's systematic review, which reported a median of 17 panel members per panel.^28^^,^^29^

Eligibility criteria for health professionals included at least 10 years of experience in specific clinical specialties, public health, and research, and have conducted studies or been involved in operational or policy aspects during previous outbreaks, epidemics, and pandemics, as identified in the literature. Detailed information on participant eligibility, including specific expertise, roles, and specialties required, can be found in the protocol.^21^ Efforts were made to include experts in various communicable diseases. Some stakeholders were recommended by the Technical Advisory Group and WHO officers.

Furthermore, we invited representatives of the civil society affiliated with specialized organizations and networks dedicated to promoting the well-being of women and families during pregnancy, childbirth, and the postnatal period, especially in the context of outbreaks or epidemics.

Finally, four international stakeholder panels were recruited: maternal and perinatal health, neonatal health, public health and emergency response, and representatives of civil society. Additional details on participant selection and recruitment are outlined in the study protocol.^21^

#### Online surveys

Participants underwent a self-screening process to confirm their eligibility, interest in participation, and commitment to engage in at least two surveys.

The first round took place between September and October 2023, and involved administering a piloted questionnaire with the complete list of outcomes identified in the systematic review. Participants had access to the systematic review report. They were asked to rate the importance of each outcome using a nine-point differential Likert scale ranging from 1 to 9, where 1 represented ‘not important at all’ and 9 ‘extremely important. Outcomes rated from 1 to 3 were classified as ‘not important’; those rated 4–6 were classified as ‘important but not critical’; and ratings of 7–9 as ‘critically important’. Additionally, the questionnaire included open-ended questions to suggest additional outcomes.

The second survey, conducted between November and December 2023, involved re-rating items subject to disagreement. Participants were provided with their own previous ratings, ratings distributions from each panel, and summaries of comments.

Both questionnaires included open-ended sections to provide comments, had explanations of medical terms, and were hosted on Delphi Manager.^30^ Participants received weekly reminders to complete each survey.

An agreement to include an outcome was defined if it was rated as critically important by at least 80% of all stakeholder panels and as not important by no more than 10%. Agreement to exclude an outcome was defined if it was rated critically important by less than 65% of participants in all stakeholder panels. If outcomes did not meet either of these criteria, they were categorized as ‘no agreement’. When no agreement was observed for an outcome in two consecutive rounds, the outcome was eliminated.

During the process, it was identified that certain outcomes were not suitable for systematic collection in all studies. Consequently, it was decided to create two categories to classify COS outcomes: “*main outcomes”*, which should be measured in all studies, and *“complementary outcomes”.* The latter refers to outcomes that are important to measure in specific types of studies (e.g., epidemiological, product development, or post-authorization surveillance), settings (depending on resource availability), or particular disease outbreaks. Nevertheless, in research on emerging pathogens, with unknown effects on maternal and neonatal health, researchers should aim to report all complementary outcomes or explain the reasons why they are not reported.

#### Consensus meetings

A purposive sample of 24 stakeholders, who completed the two online surveys and represented all panels and world regions were invited to participate in the consensus meetings.

The first meeting, held virtually on January 16 and 17, 2024, aimed to discuss included outcomes, additional outcomes suggested, and outcomes considered borderline. Outcomes were deemed borderline if, in the second survey, ≥90% of participants in at least one stakeholder panel rated the outcome as critically important. After a guided discussion, participants were asked to rate the additional and borderline outcomes.

Up to this point in the process, none of the outcomes highly rated by the civil society panel had been included, and the “Functioning and well-being” domain had no outcomes selected. COMET emphasizes including outcomes across various domains.^18^ However, it provides limited guidance in managing multistakeholder panels and the risk of diluting civil society representatives' voices.^31^^,^^32^ The RAND organization and some COS developers propose conducting independent discussions with civil society representatives, which would later be integrated in to the consensus process.^33^, ^34^, ^35^ Therefore, a virtual session was held with civil society representatives to discuss patient-oriented outcomes that were highly rated by the civil society panel. These prioritized outcomes were further discussed later by all panels.

The in-person consensus meeting took place in Geneva on February 14 and 15, 2024. It aimed to review all included outcomes, the feasibility of measuring them in all settings, and discuss outcomes’ definitions. Participants were provided with literature summaries and definition reports. Details on how the literature was reviewed and summarized are described in Supplementary File S2. Given that the International Classification of Diseases (ICD) and WHO definitions are periodically updated based on scientific evidence, participants were encouraged to prioritize these definitions whenever possible. Participants worked in small-groups and plenary discussions, and voting (when required).

### Statistics

The results for each outcome were summarized using descriptive statistics and graphically represented using histograms. A thematic analysis of the open-ended questions from the two survey rounds was conducted to identify prominent themes and key messages. Notes from group discussions consensus meetings were systematically recorded and analysed to capture and summarize the rationale behind the consensus process. The summary of discussions and the consensus reached were sent to the participants for review and approval.

### Ethics

This study was granted an exception from the WHO Ethics Review Committee, as it was determined that there was no potential for harm resulting from the conduct of this project. It was approved by the RESPIRE Research Ethics Committee in Argentina (Registry number 10357). Participants provided written consent and declare conflicts of interest before completing the first survey.

### Role of the funding source

The Bill and Melinda Gates Foundation officers had no involvement in the study design, data collection, analysis or manuscript preparation. Authors MBo, MBab and OTO, who are affiliated to WHO, contributed to design the study and in the decision to submit for publication.

## Results

### Panels and stakeholders’ characteristics

**Fig. 2:**
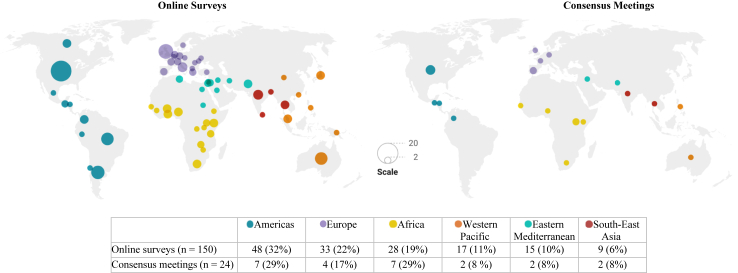
Geographical distribution of participants. Disclaimer: The boundaries and names shown and the designations used on this map do not imply official endorsement or acceptance by the World Health Organization. Note: The figure on the left represents the countries of residence of the participants from Delphi Round 1 (N = 150), grouped by WHO region. This geographic distribution was roughly maintained through Delphi Round 2 which had 141 participants (94% retention rate). Figure created with Datawrapper.

Out of 197 stakeholders invited, 159 (76%) responded to the invitation and agreed to participate, and 150 completed the first online survey. The first round ensured broad representation, with no fewer than 20 participants from each stakeholder panel, with the Maternal and Perinatal Health panel having the most participants (n = 73, 49%). Most participants were over 40 years old (85%), with “researcher” being the most frequently reported primary role (46%). However, participants reported multiple and varied roles and specialties (Table 1). In the second round, 141 participants completed the survey (attrition rate of 6%). Participants were from 60 different countries, and represented all WHO world regions (Fig. 2).^36^

**Table 1:** Characteristics of participants.

Characteristics	First online survey, n	%	Second online survey, n	%	Consensus meetings, n	%
**Total**	150		141		24	
**Stakeholder group**						
Maternal and perinatal health	73	49	71	50	7	29
Neonatal health	30	20	29	21	6	25
Public health and emergency response	26	17	23	16	5	21
Representatives of the civil society	21	14	18	13	6	25
**Gender**						
Female	99	66	93	66	18	75
Male	50	33	47	33	6	25
Prefer not to say	1	1	1	1	0	0
**Age (years)**						
30–39	22	15	20	14	2	8
40–49	51	34	48	34	6	25
50–59	47	31	43	31	11	46
≥60	29	19	29	21	5	21
Prefer not to say	1	1	1	1	0	0
**Main role**						
Researcher	69	46	65	46	9	38
Healthcare provider	43	29	43	31	9	38
Women/community representative	10	7	10	7	3	13
Health service manager	7	5	6	4	2	8
Program manager	7	5	5	4	0	0
Policy maker	6	4	4	3	0	0
Funder	4	3	4	3	0	0
Regulator	4	3	4	3	1	4
**Main specialties**^a^						
Maternal health	75	50	72	51	10	42
Epidemiology and public health	54	36	49	35	17	71
Neonatal and pediatric health	46	31	43	31	11	46
Patient advocacy	23	15	21	15	8	33
Infectious disease	18	13	18	13	4	17
Pharmacy/laboratory	8	5	7	4	2	8
Psychiatry/psychology/social work	5	3	4	3	0	0
Critical care	10	7	10	7	3	13
Other^b^	14	9	13	9	6	25

a Participants could select more than one specialty.

b Anthropology, congenital anomalies, genetics, parasitology, hematology and blood transfusion, physiology, anesthesiology; service or program manager.

The subsample of 24 stakeholders who participated in consensus meetings showcased a broad geographic representation (19 countries and all WHO regions), had diverse roles and specialties, and was well-balanced across stakeholder panels (Table 1, Fig. 2).

### Identification of outcomes

The systematic review included 440 studies conducted in 107 countries. Most of these studies were epidemiological (90·9%), with a smaller proportion related to post-authorization surveillance (8·2%) or product development (0·9%). Most studies focused on respiratory diseases (79·8% on COVID-19 and 4·1% on influenza), while a smaller proportion addressed vector-borne diseases (12·5% on Zika, 1·4% on chikungunya and 1·1% on dengue), foodborne diseases (0·5% on cholera), and haemorrhagic fever zoonosis (0·2% on Ebola, 0·2% on Rift Valley fever, and 0·2% on yellow fever). A total of 89 maternal and pregnancy outcomes and 47 neonatal outcomes were identified and included in the first round.^27^

### Delphi surveys

In the first survey, consensus was achieved to include seven maternal outcomes (*livebirth, maternal death, perinatal death, stillbirth, postpartum haemorrhage, vertical transmission, admission to intensive care unit*) and four neonatal outcomes (*neonatal death, severe disease-related outbreak disease, admission to intensive care unit, mechanical ventilation)*, and to exclude 43 maternal and seven neonatal outcomes. During this round, 75 outcomes did not reach consensus, and 21 additional outcomes were proposed by participants (Supplementary Table S1).

The second survey involved re-rating 39 maternal and 36 neonatal outcomes. Consensus was achieved to include seven maternal outcomes (*preterm birth -unspecified aetiology, spontaneous preterm birth, maternal confirmed infection, progression to severe disease, sepsis, gestational age,* and *mechanical ventilation*) and nine neonatal outcomes (*birth weight, gestational age at birth, birth asphyxia, sepsis, respiratory distress syndrome, confirmed infection -related outbreak disease, symptomatic infection, severe disease, cardiopulmonary resuscitation*). No outcomes reached consensus for exclusion, and six outcomes were identified as borderline (*fetal growth restriction, maternal symptomatic infection, thrombo-embolic event, hypertensive disorders of pregnancy, any neonatal respiratory disorder(s) and skin-to-skin contact*).

**Fig. 3:**
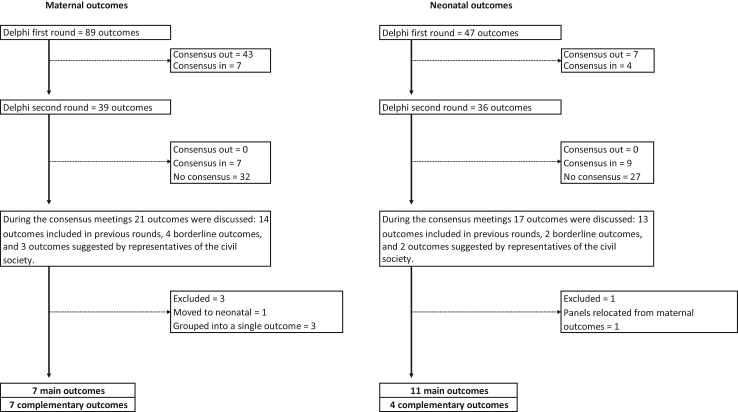
Flowchart of the outcomes selection process.

The rating distributions for each round and outcome is available in Supplementary Tables S2 and S3. The number of outcomes assessed in each round is shown in Fig. 3.

### Consensus meetings

#### Virtual meetings

During the first virtual meeting, six borderline outcomes and 21 additional outcomes were discussed and rated. Three outcomes (*cause of death*, *hypertensive disorders of pregnancy* and *maternal symptomatic infection*) reached consensus for inclusion. Subsequently, included outcomes were reviewed to discard overlaps between outcomes and ascertain their applicability across different types of studies and diseases. As a result, two outcomes were excluded: *perinatal mortality,* given the overlap with *stillbirth* and *neonatal mortality*, and *gestational age at birth* which was considered an enabling variable required to measure outcomes. Stakeholders agreed to report *low birth weight* instead of *birth weight*.

A subsequent virtual session was convened with civil society representatives to discuss outcomes related to the domain of “Functioning and well-being”. The group discussed and prioritized the following outcomes: *maternal depression (or maternal mental health)* and *gender-based violence* due to its increase in the context of epidemics; *mode of delivery* given its capacity to monitor disparities in healthcare; and *skin-to-skin contact* and *breastfeeding within the first hour*, as they might be (or need to be) discouraged due to the risk of transmission depending on the disease or the availability of scientific evidence.

#### In-person meeting

During the in-person meeting, after assessing measurement feasibility, participants agreed to include 18 main outcomes, remove one outcome (*neonatal cardiopulmonary resuscitation*), and classify 11 outcomes as complementary. Participants also agreed on outcomes’ definitions. *Gender-based violence* was moved to a post-consensus further evaluation because it was considered necessary to ensure that data collection had no inconsistencies with WHO guidelines. Box 1 presents the final COS.

**Box 1** **Final MNH-EPI-COS**

**Table:**

### Consensus meetings key discussion points

#### Main maternal outcomes

Participants agreed to consolidate mortality and vital status outcomes into a single outcome named *pregnancy outcome*, which includes *live birth, stillbirth, miscarriage/ectopic pregnancy*, and *induced abortion*. In terms of stillbirth subclassification, priority was given to distinguishing between early/late stillbirths due to their implications for practice, as opposed to categorizing them on antepartum/intrapartum or fresh/macerated, which are more susceptible to misclassification. Concerns were raised that data on *induced abortion* might not be collected in settings where elective abortion is considered illegal.

The outcome *cause(s) of death*, was incorporated into the definition of *maternal death*, emphasizing the importance of identifying, classifying, and reporting the causes of death.

The feasibility of measuring *maternal suspected symptomatic infection* and *maternal symptomatic infection* was discussed, recognizing potential limitations in systematic data collection due to resource availability, staff expertise and training, infrastructure through which to collect data, and an evolving symptom profile. It was emphasized that additional guidance is needed on how to establish a confirmed infection since it is very disease-specific.

There were discussions about redundancy between the outcomes *spontaneous or iatrogenic preterm birth* (maternal outcome), and *prematurity* (neonatal outcome). While these outcomes refer to the woman and neonate respectively, combining them requires linking maternal and neonatal records, which could impact data collection in some settings. Furthermore, measuring only prematurity would limit the data collection of labour onset, which may vary during epidemics.

#### Complementary maternal outcomes

Outcomes related to specific causes of maternal morbidity (*antepartum haemorrhage, postpartum haemorrhage, hypertensive disorders of pregnancy,* and *sepsis*), were proposed as complementary outcomes because they may not be relevant to every disease or type of study. For example, certain outcomes should be measured when the outbreak infection is suspected or confirmed to influence pathophysiological mechanisms of these outcomes (e.g., SARS-CoV-2 infection and preeclampsia). However, they may not be necessary for other diseases where such relationship is not suspected (e.g., Zika virus and preeclampsia). Certain outcomes should be considered when assessing the safety of interventions. In studies where these complementary outcomes are not being collected, the impact of the epidemic on these conditions can be assessed through mortality analysis. Finally, stakeholders highlighted that these outcomes may need to be measured if they could be confounders or the outbreak is related to a pathogen that has not yet been characterized.

The outcome *maternal admission to intensive care unit*/*special unit* generated debate about the global variability of access to intensive care and the validity of the outcome in low-resource settings. It was considered an outcome that measures the capacity to respond to emergencies and not exclusively the type of care required or the severity of the disease. Participants agreed that in settings where intensive care is available, the outcome should be reported. The outcome *maternal mechanical ventilation* was renamed as *maternal respiratory support* to include any respiratory therapy given the varying availability of respiratory support interventions in different settings. This outcome was proposed as complementary due to reporting concerns in settings with restricted availability and because it may be more relevant for respiratory diseases outbreaks.

Participants strongly agreed on the importance of measuring *maternal depression* and *anxiety*, because both, women experiencing pregnancy during infectious disease outbreak or pregnant women who have an infection may have a higher risk of experiencing these outcomes than the general population. However, concerns were raised regarding the appropriateness of conducting screening tests in settings where diagnostic and management pathways are not well established. Consequently, it was proposed to measure them in settings where systems for referral, diagnosis and management of women who screen positive are established, or could be provided in the context of research. Additionally, it might not be suitable for product development and post-authorization surveillance.

Participants unanimously emphasized the relevance of gender-based violence; however, concerns were raised due to the current lack of WHO recommendations for population-wide screening, and the substantial underreporting associated with administrative data, particularly in countries where the issue is most prevalent. In addition, this outcome was related to isolation rather than the infectious disease. Therefore, it was decided not to include it in the final list of outcomes. Nonetheless, it could be measured in epidemiological studies of future epidemics. Special emphasis was placed on mistreatment within healthcare facilities.

#### Main neonatal outcomes

A data lag in measuring *neonatal symptomatic infection* was anticipated, particularly regarding the teratogenic effects of congenital infection during an outbreak, as some pregnancies would need to reach completion before comprehensive information on effects of in-utero infection became available. Panellists suggested that samples such as amniotic fluid, umbilical cord, and placenta could be utilized to evaluate for *neonatal confirmed infection*. It was noted that disease-specific guidance should be followed to establish a confirmed infection since it varies depending on the disease. Panellists noted the importance of recognising that the neonatal symptoms of infection may differ from those observed in adults, and that any associated teratogenic effects may be characterised by features that are not predictable or related to the understood disease mechanisms of the infectious agent, or therapeutic mechanism of action of an intervention.

Similar to the maternal set, *cause(s) of death* was incorporated into the definition of *neonatal death*.

Despite anticipated challenges in the feasibility of measurement, *neonatal respiratory support* was included as a main outcome because, in contrast to *maternal respiratory support,* the need for neonatal respiratory support is far less specific to respiratory illnesses and can reflect many underlying aetiologies. The term was adapted to encompass any respiratory technique administered, acknowledging variations in available respiratory support across settings.

Overlaps between *skin-to-skin contact* and *breastfeeding in the first hour* were discussed. However, some participants argued that they are distinct because some mothers cannot, or choose not to, breastfeed, or breastfeeding might be discouraged if there is a risk of transmission through breast milk. Some participants emphasized the importance of monitoring these two outcomes during an outbreak, given that depending on the disease and scientific evidence availability, the practice could be discouraged due to the risk of transmission. Regarding *breastfeeding*, it was proposed to measure it within the first hour after birth because the timing of discharge varies significantly across setting, particularly in the context of an epidemic.^37^, ^38^, ^39^, ^40^, ^41^

#### Complementary neonatal outcomes

Neonatal morbidity such as *birth asphyxia, neonatal sepsis,* and *neonatal respiratory failure* were proposed as complementary outcomes. These outcomes should be collected when these conditions are associated with the outbreak diseases, or in research on emerging epidemic threats that have not yet been characterized. The *neonatal sepsis* definition was revised to exclude language around respiratory signs to prevent overlap with *neonatal respiratory failure* and to address feasibility concerns regarding access to blood culture. *Neonatal respiratory failure* replaced the outcome *neonatal respiratory distress* given that includes all causes of respiratory failure within the neonatal period.

The discussion around *neonatal admission to the intensive care unit* sparked a debate about global variability in access and practices leading to admission to intensive care. While some participants suggested broadening the definition to include any ward capable of offering 24-h vital care to enhance measurement feasibility, others argued for its inclusion as a complementary outcome due to practical limitations in certain regions. However, in settings where there are not significant access barriers, it should be measured given its increased validity.

*Neonatal cardio-pulmonary resuscitation* was excluded from the COS and placed in the complementary outcomes, due to its multifactorial non-specific nature.

Agreed main and complementary outcomes definitions, and discussion remarks are presented in Table 2.

**Table 2:** Final COS outcomes definitions and remarks.

Domain and outcome	Outcome definition and measurement	Notes/remarks
**Maternal main outcomes**		
**Mortality/vital status**		
Pregnancy outcome	Results of conception and pregnancy, measured as: Live birth is an outcome of pregnancy, irrespective of the duration/gestation, where the newborn breathes or shows any other evidence of life–e.g., beating of the heart, pulsation of the umbilical cord or definite movement of voluntary muscles– whether the umbilical cord has been cut or the placenta is attached. Stillbirth is the complete expulsion or extraction from a woman of a foetus, following its death prior to the complete expulsion or extraction, at 22 or more completed weeks of gestation. When information on gestational age is unavailable use birthweight 500 g or more as criteria. Miscarriage (also known as unintentional abortion or spontaneous abortion) is a spontaneous loss of pregnancy (i.e., embryo or foetus) before 22 completed weeks of gestation. When information on gestational age is not available, use birth weight of less than 500 g as a criterion. Induced abortion is a complete expulsion or extraction from a woman of an embryo or a foetus (irrespective of the duration of the pregnancy) following a deliberate interruption of an ongoing pregnancy, which is not intended to result in a live birth.	This definition may be variably interpreted in different settings according to the gestational age and/or weight of the baby, which may affect comparability. There is a need for standardization of its use across settings and for consistency with definitions of miscarriage and stillbirth. Stillbirths differ from induced abortion. Gestational age (GA) should be used whenever possible, with weight being utilized only in settings where accurately estimating GA is not feasible. It can be reported as early (22-27w–or 500–999 g) and late (≥28w–or 1000 g) stillbirths. A cut-off of 20 or 22 weeks for the definition was selected to stay consistent with WHO guidance given international variability. *Ectopic pregnancy* is proposed to be reported together with *Miscarriage*. Data on induced abortions may not be routinely available in settings where there are restrictive abortion laws. It may also be misclassified as miscarriages or stillbirths.
Maternal death	Maternal death is defined as the death of a woman while pregnant or within 42 days of termination of pregnancy, irrespective of the duration and site of the pregnancy, from any cause related to or aggravated by the pregnancy or its management, but not from unintentional or incidental causes.	Leading cause(s) of death should be reported following the ICD-MM classification system (ICD-MM). The cause(s) of death must be specific, so that the impact of the epidemic is not only measured through variations in the relative weight of all indirect causes, but the specific deaths rate attributable to disease outbreak.
**Maternal infection (related outbreak disease)**		
Maternal suspected symptomatic infection (related outbreak disease)	A woman who meets clinical criteria (signs and symptoms) for the disease under investigation while pregnant or within 42 days after birth.	Feasibility may be limited by staff training, data collection infrastructure and evolving case definitions. It is recommended to use WHO case definitions, which are regularly updated and allow collating data. This should be reported regardless of vital status
Maternal confirmed infection (related outbreak disease)	A woman with a positive and confirmatory laboratory or pathology result while pregnant or within 42 days of end of pregnancy, regardless of clinical OR epidemiological criteria for the disease under investigation, and vital status.	Pathology results refer to recognized pathogen identified using a validated method. There is a risk of under-reporting because of lack of access to testing. It is recommended to use WHO case definitions, which are regularly updated and allow collating data.
Maternal severe disease (related outbreak disease)	A woman presenting while pregnant or within 42 days of end of pregnancy with illness (specific to the disease under investigation) that results in acute physiological instability (abnormal physiological parameters or vital organ dysfunction or failure) or a clinical support requirement (such as hospitalization, intensive care or high-dependency unit or time-sensitive intervention) to prevent clinical deterioration, disability or death.	Some women with severe cases cannot access care and die. Although maternal death is a separate outcome, it must be part of any assessment of disease severity. Near miss criteria can be used to measure instability.
**Labour and delivery characteristics**		
Spontaneous/iatrogenic preterm birth	Preterm is defined as babies born alive before 37 weeks of pregnancy are completed Spontaneous preterm birth is defined as the spontaneous onset of labour and delivery of a live born between 22 and 36 completed weeks. Iatrogenic preterm birth (also known as medically indicated for foetal and/or maternal interest), is defined as a live born between 22 and 36 completed weeks after induction of labour or a caesarean before labour.	While there is overlap with *prematurity* (neonatal outcome), this outcome monitors the impact of the epidemic on spontaneous preterm birth and clinical practice. In settings where maternal and neonatal registries cannot be linked, prematurity could be measured, but it might be challenging to determine whether labour onset was spontaneous, induced, or due to a planned caesarean section.
Mode of birth	Parturition of a newborn from the uterus via spontaneous vaginal birth, assisted vaginal birth (vacuum or forceps), elective caesarean section, or emergency caesarean section.	
**Maternal complementary outcomes**		
**Morbidity**		
Antepartum haemorrhage	Vaginal bleeding after 22 weeks of pregnancy or during labour before giving birth.	Pathologic aetiologies include placenta previa, placenta accreta, vasa previa, abruptio placentae, and uterine rupture. This outcome should be measured if it known to be (or might be) associated with outbreak disease under investigation, or during emerging epidemic threats that have not yet been characterized.
Postpartum haemorrhage	A blood loss of 500 ml or more within 24 h after birth.	This condition is caused by uterine atony, trauma, retained placenta, or coagulopathy. Visually estimated blood increase misclassification. A calibrated drape or other device should be considered to measure blood loss in all births. This outcome should be measured if it is (or might be) associated with outbreak disease under investigation or during emerging epidemic threats that have not yet been characterized.
Hypertensive disorders of pregnancy	A hypertensive disorder newly diagnosed after 20 weeks’ gestation or before 1 week postpartum, characterized by systolic blood pressure greater than 140 mmHg and/or a diastolic blood pressure greater or equal to 90 mmHg on two occasions, 4 h or more apart.	Laboratory or point of care confirmation, where feasible, should include the evaluation of hepatic, renal or haematological changes, and a urine test. In situations where healthcare services are overwhelmed during an epidemic, the second blood pressure assessment may be performed≥1 h apart from the first one. This outcome should be measured if it is (or might be) associated with outbreak disease under investigation during emerging epidemic threats that have not yet been characterized.
Maternal sepsis	A life-threatening condition defined as organ dysfunction resulting from infection during pregnancy, childbirth, post-abortion, or post-partum period.	Organ dysfunction is measured using specific scores (e.g., SOFA), that usually require laboratory. In low resource settings, near miss criteria or obstetric early warning systems could be considered as a proxy for assessing organ dysfunction.
**Delivery of care**		
Maternal admission to intensive care unit/special unit	Admission to an intensive care unit or a unit that provides 24-h monitoring and vital support at any point during pregnancy through 42 days after pregnancy (postpartum, post-abortion/miscarriage) for any obstetric indication, outbreak disease or other non-obstetric indications.	The definition encompasses a range of special care units (e.g., high dependence unit and special care unit) providing continuous monitoring and vital support. Research conducted in settings capable of providing critical care without substantial access problems should report this outcome.
Maternal respiratory support	Any respiratory support technique with any level of pressure via any interface, during pregnancy, -labour and 42 days after pregnancy, not related to anaesthesia during delivery (postpartum/postabortion).	This outcome should be measured systematically in settings where access to this intervention is not an issue (where the results capture the real need) and when is relevant to the type of disease (e.g., respiratory infections). In settings where access to mechanical ventilation is not restricted, this outcome could be further categorized into specific respiratory support types to better reflect disease severity.
**Maternal Functioning**		
Maternal symptoms of depression and anxiety	Depressive disorders are characterized by sadness, loss of interest or pleasure, feelings of guilt or low self-worth, disturbed sleep or appetite, feelings of tiredness, and poor concentration. Anxiety disorders refer to a group of mental disorders characterized by feelings of anxiety and fear, including generalized anxiety disorder (GAD), panic disorder, phobias, social anxiety disorder, obsessive-compulsive disorder (OCD) and post-traumatic stress disorder (PTSD).	Depression can be long lasting or recurrent, substantially impairing an individual's ability to function at work/school or cope with daily life. Severe depression can lead to suicide. Screening could be conducted during the antenatal or postnatal period (six weeks after birth). Symptoms of depression and anxiety can be identified by administering self-administered validated tools, such as the Edinburgh Postnatal Depression Scale (EPDS) or Patient Health Questionnaire (PHQ-9). A total score of≥11 of the EPDS might indicate depressive or anxiety symptoms. This outcome is complementary because it should only be measured when systems for referral, diagnosis and management of women who screen positive are established to ensure adequate follow-up. On the other hand, not all types of studies should include this outcome (e.g., product development and post-authorization surveillance).
**Neonatal main outcomes**		
**Mortality/vital status**		
Neonatal death	A death following a live birth, with 22 or more completed weeks of gestation (or 500 g or more) occurring from time of birth to 28 days after birth (note day 1 is the day of birth).	The definition includes early neonatal deaths occurring from birth through day 7 after birth, and late neonatal death from day 8–28 days after birth. It should be reported as overall and disaggregated rates. Where possible the leading cause(s) of deaths for newborns should be reported following ICD-PM (ICD-PM definition)
**Neonatal infection**		
Neonatal symptomatic infection (related outbreak disease)	A newborn (1–28 days after birth) who meets clinical criteria (signs and symptoms) associated with a disease under investigation, including antenatal and postnatal infection.	Case definitions for neonates differ to adults. It is recommended to use WHO case definitions, which are regularly updated and allow collating data. Feasibility may be limited by staff training, data collection infrastructure and evolving case definitions.
Neonatal confirmed infection (related outbreak disease)	A newborn (1–28 days after birth) with a positive and confirmatory laboratory or pathology result (recognized pathogen identified using a validated method), regardless of clinical criteria OR epidemiological criteria (related outbreak in investigation), antenatal or postnatal acquisition, and vital status.	Maternal infection should not automatically be assumed to reflect foetal infection. When available, pathology (amniotic fluid, umbilical cord, placenta) can be used to confirm an infection. Disease-specific guidance will be needed on how to establish a confirmed infection (e.g., sample collection, testing options). It is recommended to use WHO case definitions, which are regularly updated and allow collating data. Feasibility may be limited by staff training, equipment, supplies, data collection infrastructure and evolving case definitions.
Neonatal severe/critical disease (related outbreak disease)	A newborn (1–28 days) with an antenatally or postnatally acquired illness (specific to the outbreak under investigation) that results in acute physiological instability (abnormal physiological parameters or vital organ dysfunction or failure) or a clinical support requirement (such as hospitalization, admission to NICU, intensive care or high-dependency unit or time-sensitive intervention) to prevent further clinical deterioration, disability, or death.	Disease-specific guidance will be needed on how to establish a severe/critical disease. It is recommended to use WHO case definitions, which are regularly updated and allow collating data. Feasibility may be limited by staff training, equipment, supplies, data collection infrastructure and evolving case definitions.
Vertical transmission	Transmission of pathogen from a parent to the foetus or baby during pregnancy (in utero), intrapartum by exposure to blood and secretions, and by exposure after birth via breast milk.	Although most infections can be transmitted from mother to foetus/baby, there is a small but not negligible possibility of disease transmission through seminal fluid.
**Morbidity**		
Low birthweight	A live born with weight less than 2500 g at birth. Sub-categories include: o Extremely low birth weight (<1000 g) o Very low birth weight (1000–1499 g) o Low birth weight (1500–2499 g)	It should only measure among neonates (live born) and it should be reported as overall and disaggregated rates.
Prematurity	Babies born alive greater than or equal to 22 + 0 and less than 37 completed weeks of gestation. Sub-categories include: o Extremely preterm (22 + 0 w to 27 + 6) o Very preterm (28 + 0 w to 31 + 6) o Moderately preterm (32 + 0 w to 33 + 6) o Late preterm (from 34 + 0 weeks to 36 + 6)	It should be reported as overall and disaggregated rates.
Any congenital anomaly	Any structural or functional anomalies that develop in utero, and may be identified before, at birth, or after birth.	The definition encompasses external, internal, and developmental anomalies. Congenital anomalies should be classified and reported following WHO/ICBDSR/CDC birth defects surveillance manual and ICD-11 version.
**Delivery of care**		
Neonatal respiratory support	Any respiratory support technique to a neonate between day 1 and 28 after birth with any level of pressure via any interface, not related to anaesthesia.	The definition includes any respiratory support technique (e.g., mask, cannula, nasal prong O2, CPAP, high-flow treatment) administered at any pressure in response to concerns around varying degrees of respiratory support available in different healthcare settings.
Skin-to-skin contact during the first hour after birth	A newborn without complications is kept in skin-to-skin contact with her/his mother, placed prone on the mother's abdomen or chest in direct ventral-to-ventral skin-to-skin contact for at least an hour or until after the first feed.	The baby should be kept dry and warm with a blanket or the mother's gown covering the baby's back, and the infant may have worn a diaper or cap.
Breastfeeding within 1 h of birth	Newborn who is put to the breast during as soon as possible after birth and within 1 h of birth, when they are clinically stable, and the mother and baby are ready.	Early initiation of breastfeeding does not require that the infant suckled at the breast or that milk was transferred from breast to infant. It represents the practice of putting the baby to breast within the first hour, which is related to a number of positive outcomes including reduced mortality and exclusive breastfeeding (47).
**Neonatal complementary outcomes**		
**Morbidity**		
Birth asphyxia	Birth asphyxia is the inability of a newborn to start and sustain breathing immediately after birth, characterized by a low Apgar score and/or metabolic acidosis. It is classified in: o Mild and moderate: Normal respiration not established within 1 min, but heart rate 100 per minute or above, some muscle tone present, some response to stimulation. Apgar score 4–7 at 5 min and/or pH < 7.2 on the umbilical cord arterial blood sample o Severe: Pulse less than 100 per minute at birth and falling or steady, respiration absent or gasping, colour poor, tone absent. Apgar score 0–3 at 5 min. And/or also characterized by profound metabolic acidosis, pH < 7.0 on the umbilical cord arterial blood sample.	This definition increase feasibility for data collection in all resource settings, allowing for using the Apgar score or diagnosis of acidosis in umbilical cord blood. This condition may not be relevant to all disease outbreaks. This outcome should be measured if it is (or might be) associated with outbreak diseases or in research on emerging epidemic threats that have not yet been characterized.
Neonatal sepsis	A condition affecting foetuses or newborns, that is (or suspected to be) caused by a maternal infection (acquired in utero or during birth) with a bacterial, viral, fungal, or parasitic source.	The definition includes “suspected” cases given feasibility concerns around access to blood culture. This outcome should be measured if it is (or might be) associated with outbreak diseases or in research on emerging epidemic threats that have not yet been characterized.
Neonatal respiratory failure	Acute onset of respiratory failure in a newborn (any gestational age or birthweight), in the first 28 days after birth, from any cause. It is recognized as one or more signs of increased work of breathing (such as tachypnoea, nasal flaring, chest retractions, or grunting) with or without cyanosis.	This definition encompasses all causes of respiratory failure irrespective of the cause. This outcome should be measured if it is (or might be) associated with outbreak diseases or in research on emerging epidemic threats that have not yet been characterized.
**Delivery of care**		
Neonatal admission to the intensive care unit/other special units	Admission of a neonate in the first 28 days after birth to an intensive care unit or a unit that provides 24-h monitoring and vital support for any indications.	The definition has been modified to encompass a range of special care units providing care to all critically ill neonates across various care settings. It includes any ward capable of offering 24-h vital care. Research conducted in settings capable of providing this type of care critical care without substantial access problems should report this outcome.

Notes: GA, gestational age; w, weeks; g, grams; NICU, Neonatal Intensive Care Unit.

## Discussion

A consensus on a COS for maternal and neonatal health research and surveillance of emerging and ongoing epidemic threats was obtained in an international modified Delphi study. The COS consists of seven main and seven complementary maternal outcomes, along with 11 main and four complementary neonatal outcomes. Definitions were also agreed for all outcomes.

Currently, there are no other published COS on maternal and neonatal health specifically designed for application in the context of infectious disease outbreaks and epidemics. Our findings align with established broader outcome sets on maternal and neonatal health research as well as those focusing solely on certain pregnancy conditions, vaccine safety monitoring during pregnancy, and COVID-19 research.^20^^,^^42^, ^43^, ^44^, ^45^, ^46^ These COSs include a range of outcomes reflecting pregnancy outcomes, maternal and neonatal mortality, health and morbidity, as well as social functioning and well-being.^46^ They include outcomes such as stillbirth, preterm birth, mental health, mother-infant bonding, confidence, breastfeeding, maternal death, hypertension, postpartum haemorrhage, maternal and neonatal admission to intensive care, maternal and neonatal respiratory support, and congenital anomalies.^43^, ^44^, ^45^^,^^47^

In relation to outcomes closely associated with infectious disease outbreaks, a minimal common outcome measure set for COVID-19 clinical research aligns with our approach in adopting outcomes related to viral burden, survival, and clinical progression. Pregnancy outcomes included preterm delivery, miscarriage, and fetal status.^42^

Researchers are encouraged to collect all main outcomes from the COS and, when applicable, complementary outcomes. If they choose not to measure certain outcomes, they should provide a justification for this decision. We recognize that certain outcomes, although essential, may be difficult to be measured, particularly during wide outbreaks and shortage of resources. For instance vertical transmission may require several tests and different techniques and impose a significant burden on providers.^48^ Other outcomes are easy to measure but may be considered “weak” because they rely on criteria non universally shared (e.g., admission to intensive care). In such cases, efforts should be deployed at a medical specialty level to harmonise criteria as this may also have an impact in terms of public health.^49^ Such standardization may also benefit responses to other outbreaks not involving mother-to-child transmission.^50^

Overall, these examples and the experience that will be accumulated could inform future adjustments for this COS. Also, additional outcomes should be considered depending on the research question. Certain additional outcomes can be reported without the need to collect additional variables. For instance, weight-for-gestational-age can be derived from the same enabling terms (birth weight and gestational age) used to report low birth weight and prematurity. The results of this systematic review, which reported outcomes across different types of research studies and diseases could facilitate the selection of additional specific outcomes.

Finally, definitions were agreed upon with consideration of their applicability across all settings, including those with limited resources. However, some outcome definitions are aspirational in certain settings. For instance, defining stillbirth to include all fetal deaths from 22 weeks of gestational age, instead of the 28-week threshold often used for statistical purposes, poses a challenge.

This study has several strengths. First, the methodology was defined *a priori* based on the guidelines of the COMET Initiative, and the development of the core outcome set followed a rigorous multistage approach. Second, it included diverse perspectives from international key stakeholders, including representatives from civil society. Participants came from a wide array of countries with varying income levels, ensuring the relevance and applicability of the developed COS across diverse settings. Other strengths of the study include the low attrition follow-up rate, and the diversity of domains, such as patient-relevant outcomes and those related to service delivery. We also advanced the definitions of each outcome within the COS. Finally, this COS is not limited to experimental studies, but can also be applied to epidemiological research.

However, our study has some limitations. There were imbalances in the size of the panels, although this was compensated for the equitable weight ascribed to each of the four panels for decision-making, independent of its size. No participants with expertise in mental health were invited to the face-to-face meeting, as a consequence the consensus on the definitions of “depression and anxiety” and the suggestions on measurement instruments derived from published WHO recommendations. Additionally, the outcomes initially identified through the systematic review were primarily derived from epidemiological studies in the context of COVID-19; and the search was conducted from 2015 onwards, which may have led to the omission of outcomes from earlier epidemics. It is possible that we missed outcomes reported in other languages and smaller studies, specifically those with fewer than 50 participants (e.g. mpox and listeria outbreaks). Nevertheless, studies covering a range of diseases with various modes of transmission were included. We did not conduct a qualitative study to identify outcomes that were not documented in the literature. This limitation was mitigated by the participation of representatives of civil society and asking to propose additional outcomes. Finally, although the methodology is well described, the published evidence supporting it is limited, including the agreement criteria; and final decisions were made during consensus meetings. We attempted to address this limitation by following robust methodological standards. However, a necessary next step is to evaluate the acceptability of this COS on a larger scale.

This COS, developed through a robust methodology with an international multidisciplinary effort, will serve to standardize outcome selection, collection, and reporting in maternal and neonatal health research and surveillance during epidemics. We suggest that researchers conducting epidemiological studies, product development, and post-authorization surveillance report main COS outcomes as a minimum, along with complementary and additional outcomes of interest.

Further research is needed to identify stakeholders’ acceptability of the COS and validating them to facilitate the adoption of the COS and ensure its generalizability, credibility, and usability. It will be important to develop standardized data collection tools that include essential data elements to facilitate implementation. Additionally, feasibility assessments in diverse settings are required, including pilot testing of data collection in real-time outbreak scenarios, to identify potential barriers to data collection and reporting. This is especially important for observational studies, as most evaluation of barriers to the use of COS have been conducted in the context of experimental studies.

## Contributors

MBo and OTO conceived the idea and contributed to funding acquisition. VP, MBo and MBer contributed to conceptualization and methodology; VP and KK developed the protocol with input from MBo, MBer, JPA, MBel, MK and FM. VP, KK, MBo, MBer, MBa coordinated the project. MBo, MBer, MBa and VP managed resources. MBa, AMaz, VP, VO, FS, MBer, SC, ALMar, and KK conducted the systematic review and systematic searches in the literature. JA, HB, KB, OB, CC, DDL, SD, CE, MFEV, RE, EK, CRK, MK, SL, MML, SM, LM, RMM, RMB, FMR, UO, PO, DAS, TS, LY, MY, NZ, OTO, MBer, MBo, JPA, MBel, KK and VP participated in the consensus and provided input at various stages of the project. Data were curated and analyzed by VP, KK, JPA, and MBel. VP, KK, JPA, MBel, MBer, and MBo wrote the manuscript with input from all co-authors, and AMar and SC contributed to data visualization, tables and figures. All authors read and approved the final version of the manuscript, had full access to all the data, and are responsible for the decision to submit it for publication. This paper reflects the views of the named authors, and not the views of their organisations, or the views of UNDP-UNFPA-UNICEF-WHO-World Bank Special Programme of Research, Development, and Research Training in Human Reproduction or WHO.

## Data sharing statement

Data are available on reasonable request. Individual participant data that was collected throughout the research process is not available to others. De-identified participant data was aggregated for analysis and presented in an anonymized format. Extensive data are presented in the supplementary materials.

## Editor note

The Lancet Group takes a neutral position with respect to territorial claims in published maps and institutional affiliations.

## Declaration of interests

MK disclosed receiving grants from the National Institute for Health Research and the Healthcare Quality Improvement Programme, both awarded to their institution. SM disclosed serving as a member of the European Foundation for the Care of Newborn Infants (EFCNI), holding positions on both the Trustee Board and the Executive Board. These roles are unpaid. FM disclosed receiving a grant or contract from Pfizer for COVID-19 vaccine research (paid to her institution) and serving on a Data Safety Monitoring Board/Advisory Board for Pfizer and Moderna concerning RSV and other vaccines. LY disclosed receiving a five-year grant from IMI ConcePTION, which included part-time salary support to undertake project-related work as part of her job plan. Additionally, she provided consultancy services for Sanofi Genzyme South Africa on two occasions: one concerning genetic testing in Gaucher's Disease and the other involving a conference lecture on genetic testing in cardiac clinics. CE disclosed that their flight and per diem expenses for attending the in-person consensus meeting were covered by the project, while RE disclosed receiving payment for attending the consensus meeting. MBon and OTO disclosed receiving support for the present manuscript from the Bill & Melinda Gates Foundation through funding provided to their organization (WHO). All other authors declare no competing interests.
