# Supplementary material for: A core outcome set for maternal and neonatal health research and surveillance of emerging and ongoing epidemic threats (MNH-EPI-COS): a modified Delphi-based international consensus
Source: eClinicalMedicine. 2025 Jan 15;80:103025. doi: 10.1016/j.eclinm.2024.103025 (PMC11786750; doi:10.1016/j.eclinm.2024.103025)
Supplement: Supplementary Files and Tables [file mmc1.docx]

**A core outcome set for maternal and neonatal health research and surveillance of emerging and ongoing epidemic threats (MNH-EPI-COS): a modified Delphi-based international consensus**

**Supplementary materials**

[Supplementary File S1. Domains and subdomains used to classify outcomes 2](#_Toc184734864)

[Supplementary File S2. Methods additional information 3](#_Toc184734865)

[Supplementary File S3: Search strategies used in Pubmed to identify publications evaluating the feasibility of collecting and reporting each included outcome 5](#_Toc184734866)

[Supplementary File S4. Delphi Surveys Participants 10](#_Toc184734867)

[Supplementary File S5. Consensus meetings list of participants 13](#_Toc184734868)

[Table S1. Additional outcomes suggested by participants 16](#_Toc184734869)

[Table S2. Results from Round 1 and 2. Maternal outcomes 17](#_Toc184734870)

[Table S3· Results from Round 1 and 2· Neonatal outcomes 21](#_Toc184734871)

# Supplementary File S1. Domains and subdomains used to classify outcomes

This box represents the classification we used for domains and subdomains, separately for maternal and neonatal outcomes, using a simplified domain classification based on the last recommendation of COMET 2018.^1^

Maternal and pregnancy-related outcomes:

a) Mortality / vital status,

b) Physiological/clinical:

*Subdomains:* maternal morbidity, maternal morbidity not related to pregnancy (by system), placenta-related outcomes, infection and infestation outcomes, labour and delivery characteristics, foetal outcomes

c) Functioning:

*Subdomains:* Social functioning, Emotional functioning/well-being, Cognitive functioning, and Delivery of care (Obstetric management, Preventive and Therapeutic intervention related to an outbreak, Clinical Management).

Neonatal outcomes

a) Mortality / vital status,

b) Physiological/clinical:

*Subdomains:* Physiological/clinical, Neonatal outcomes, Neonatal morbidity, and Infection and infestation outcomes

c) Functioning (delivery of care).

# Supplementary File S2. Methods additional information

**Civil society representatives’ involvement**

To ensure civil society representatives could fully engage during the online surveys, we provided information sheets written in plain language to minimize barriers to understanding.

Regarding the discussion and consensus on the definitions, organizers anticipated that civil society representatives would not actively engage in discussions about the technical definitions, although they played a significant role in the broader consensus process for the final COS. However, four of the six civil society representatives had a clinical or public health background and were able to participate in the discussion of definitions.

**Methods for mapping definitions and measurement instruments, and summarizing data collection feasibility for included outcomes**

The final expert consensus meeting included, in addition to the COS finalization, discussions on outcome definitions, measurement instruments for patient-reported outcomes, and the feasibility of data collection for each outcome. Experts received summary reports that included key definitions along with their sources and publication dates, details of measurement instruments with psychometric characteristics, and feasibility summaries on data collection. The reports specifically highlighted feasibility findings from studies conducted in low- and middle-income countries. The methodology used to review the literature is presented below.

1. **Definitions mapping**

We initially consulted our systematic review database to identify definitions used by researchers. This review revealed significant variability, with authors frequently referencing definitions from established sources (e.g., ICD-11, WHO, or professional associations) and occasionally adapting them to specific contexts. Consequently, we determined that the criteria for identifying definitions would require a more rigorous approach, and developed through a systematic expert consensus process.

We searched the grey literature, by consulting the following organizations websites: ICD-11, WHO, GAIA/Brighton collaboration, World Bank, UNICEF, FIGO, NIH, CDC, NICE, RCOG, ACOG, ICHI and Eurostat. When multiple definitions were found from the same organization, we prioritized the most recent. Additionally, we reviewed all COS publications that included maternal, pregnancy, and neonatal outcomes to identify any consensus-based definitions.

1. **Measurement instruments for patient reported outcomes**

To identify measurement instruments for the included outcomes that required patient-reported assessments, we consulted two established questionnaire databases: the Consensus-based Standards for the Selection of Health Measurement Instruments (COSMIN) and Health and Psychosocial Instruments (HaPI) via EBSCO. From these sources, we extracted all relevant instruments along with their evaluations regarding psychometric properties, including reliability and validity.

1. **Systematic literature search on feasibility of outcomes of data collection**

A systematic literature search was conducted to identify studies reporting on the feasibility of data collection for each outcome included in the COS. An experienced librarian developed the search strategy (see Supplementary File S3). We searched PubMed, the Repository Maternal and Child Survival Program/USAID and studies using GAIA outcomes and case definitions. Publications were screened, and data extracted by a single reviewer. Summaries of the evidence, with a particular focus on data collection feasibility in low- and middle-income countries, were prepared and included in reports reviewed by participants during the consensus meeting.

# Supplementary File S3: Search strategies used in Pubmed to identify publications evaluating the feasibility of collecting and reporting each included outcome

*Maternal outcomes*

**Live Birth**

(Feasibility[tiab] OR Barrier*[tiab] OR Facilita*[tiab]) AND (**Live Birth[Mesh] OR Live Birth*[tiab])** AND (**Data Collection[Mesh])** AND (**Population Surveillance[Mesh] OR** Surveillance[tiab] OR Information System*[tiab] OR Registry[tiab])

**Stillbirth**

(Feasibility[tiab] OR Barrier*[tiab] OR Facilita*[tiab]) AND (**Stillbirth[Mesh] OR Stillbirth[tiab])** AND (**Data Collection[Mesh])** AND (**Population Surveillance[Mesh] OR** Surveillance[tiab] OR Information System*[tiab] OR Registry[tiab])

**Miscarriage**

(Feasibility[tiab] OR Barrier*[tiab] OR Facilita*[tiab]) AND (**Abortion, Spontaneous[Mesh] OR Miscarriage[tiab] OR Spontaneous Abort*[tiab] OR Pregnancy Loss[tiab])** AND (**Data Collection[Mesh])** AND (**Population Surveillance[Mesh] OR** Surveillance[tiab] OR Information System*[tiab] OR Registry[tiab])

**Induced Abortion**

(Feasibility[tiab] OR Barrier*[tiab] OR Facilita*[tiab]) AND (**Abortion, Induced[Mesh] OR Induced Abort*[tiab] OR Legal Abort*[tiab] OR Request Abort*[tiab])** AND (**Data Collection[Mesh])** AND (**Population Surveillance[Mesh] OR** Surveillance[tiab] OR Information System*[tiab] OR Registry[tiab])

**Maternal Death**

(Feasibility[tiab] OR Barrier*[tiab] OR Facilita*[tiab]) AND (**Maternal Death[Mesh] OR Maternal Death*[tiab])** AND (**Data Collection[Mesh])** AND (**Population Surveillance[Mesh] OR** Surveillance[tiab] OR Information System*[tiab] OR Registry[tiab])

**Maternal Infection (symptomatic and confirmed) / Sepsis**

(Feasibility[tiab] OR Barrier*[tiab] OR Facilita*[tiab]) AND (**Puerperal Infection[Mesh] OR Maternal Infection*[tiab] OR Puerperal Infection*[tiab] OR Postpartum Sepsis[tiab] OR Postpartum Infection*[tiab] OR Puerperal Sepsis[tiab])** AND (**Data Collection[Mesh])** AND (**Population Surveillance[Mesh] OR** Surveillance[tiab] OR Information System*[tiab] OR Registry[tiab])

**Severe/critical disease (related outbreak disease)**

(Feasibility[tiab] OR Barrier*[tiab] OR Facilita*[tiab]) AND (**Epidemics[Mesh] OR Epidemic*[tiab] OR Pandemi*[tiab] OR Outbreak*[tiab])** AND (**Data Collection[Mesh])** AND (**Population Surveillance[Mesh] OR** Surveillance[tiab] OR Information System*[tiab] OR Registry[tiab]) AND (Pregnancy[Mesh] OR Pregnan*[tiab] OR Maternal[tiab])

**Preterm Birth**

(Feasibility[tiab] OR Barrier*[tiab] OR Facilita*[tiab]) AND (**Premature Birth[Mesh] OR Preterm Birth*[tiab] OR Premature Birth*[tiab])** AND (**Data Collection[Mesh])** AND (**Population Surveillance[Mesh] OR** Surveillance[tiab] OR Information System*[tiab] OR Registry[tiab])

**Maternal admission to intensive care unit**

(Feasibility[tiab] OR Barrier*[tiab] OR Facilita*[tiab]) AND (**Critical Care[Mesh] OR Critical Care*[tiab] OR Intensive Care*[tiab] OR Intensive Care Units[Mesh] OR ICU[tiab])** AND (**Data Collection[Mesh])** AND (**Population Surveillance[Mesh] OR** Surveillance[tiab] OR Information System*[tiab] OR Registry[tiab]) AND (Pregnancy[Mesh] OR Pregnan*[tiab] OR Maternal[tiab])

**Maternal mechanical ventilation**

(Feasibility[tiab] OR Barrier*[tiab] OR Facilita*[tiab]) AND (**Respiration, Artificial[Mesh] OR Mechanical Ventilat*[tiab] OR Artificial Respirat*[tiab] OR Mechanical Respirat*[tiab])** AND (**Data Collection[Mesh])** AND (**Population Surveillance[Mesh] OR** Surveillance[tiab] OR Information System*[tiab] OR Registry[tiab]) AND (Pregnancy[Mesh] OR Pregnan*[tiab] OR Maternal[tiab])

**Antepartum haemorrhage**

(Feasibility[tiab] OR Barrier*[tiab] OR Facilita*[tiab]) AND (Antepartum Haemorrhage**[tiab] OR Antepartum Bleed*[tiab] OR Spotting*[tiab] OR “Pregnancy** Haemorrhage”[tiab:~3]**)** AND (**Data Collection[Mesh])** AND (**Population Surveillance[Mesh] OR** Surveillance[tiab] OR Information System*[tiab] OR Registry[tiab]) AND (Pregnancy[Mesh] OR Pregnan*[tiab] OR Maternal[tiab])

**Postpartum Hemorrhage**

(Feasibility[tiab] OR Barrier*[tiab] OR Facilita*[tiab]) AND (**Postpartum Hemorrhage[Mesh] OR Postpartum Hemorrhage*[tiab] OR PPH[tiab])** AND (**Data Collection[Mesh])** AND (**Population Surveillance[Mesh] OR** Surveillance[tiab] OR Information System*[tiab] OR Registry[tiab])

**Hypertensive disorders of pregnancy**

(Feasibility[tiab] OR Barrier*[tiab] OR Facilita*[tiab]) AND (**Hypertension, Pregnancy-Induced[Mesh] OR Gestational Hypertens*[tiab] OR “Pregnancy Hypertension”[tiab:~3] OR Eclampsia[tiab] OR Pre-Eclampsia[tiab] OR Preclampsia[tiab] OR Preeclampsia[tiab] OR Hypertensive Disorder*[tiab])** AND (**Data Collection[Mesh])** AND (**Population Surveillance[Mesh] OR** Surveillance[tiab] OR Information System*[tiab] OR Registry[tiab])

**Gestational age at delivery**

(Feasibility[tiab] OR Barrier*[tiab] OR Facilita*[tiab]) AND (**Gestational Age[Mesh] OR Gestational Age*[tiab] OR Fetal Age*[tiab] OR Fetal Maturity[tiab] OR Fetal Development[tiab])** AND (**Data Collection[Mesh])** AND (**Population Surveillance[Mesh] OR** Surveillance[tiab] OR Information System*[tiab] OR Registry[tiab])

**Violence**

(Feasibility[tiab] OR Barrier*[tiab] OR Facilita*[tiab]) AND (Violence[Mesh:noexp] OR Domestic Violence**[Mesh] OR Domestic Violence[tiab] OR Spouse Abuse*[tiab] OR Gender-Based Violence[Mesh] OR Gender-Based Violence[tiab] OR Intimate Partner Violence[Mesh] OR Partner Violence[tiab])** AND (**Data Collection[Mesh])** AND (**Population Surveillance[Mesh] OR** Surveillance[tiab] OR Information System*[tiab] OR Registry[tiab]) AND (Pregnancy[Mesh] OR Pregnan*[tiab] OR Maternal[tiab])

**Depression / Peripartum psiquiatric disorders**

(Feasibility[tiab] OR Barrier*[tiab] OR Facilita*[tiab]) AND (**Depression, Postpartum[Mesh] OR Post-Partum Depression[tiab] OR Postpartum Depression[tiab] OR Postnatal Depression[tiab] OR Psychiatric*[tiab] OR Psychologic*[tiab] OR Mood Disorder*[tiab])** AND (**Data Collection[Mesh])** AND (**Population Surveillance[Mesh] OR** Surveillance[tiab] OR Information System*[tiab] OR Registry[tiab]) AND (Pregnancy[Mesh] OR Pregnan*[tiab] OR Maternal[tiab])

**Mode of delivery**

(Feasibility[tiab] OR Barrier*[tiab] OR Facilita*[tiab]) AND (**Delivery, Obstetric[Mesh] OR Parturition[Mesh] OR Natural Birth[tiab] OR Natural Childbirth[tiab] OR Vaginal Birth[tiab] OR Vaginal Delivery[tiab] OR Cesarean[tiab] OR C-Section*[tiab] Psychiatric*[tiab] OR Labor[tiab] OR Episiotom*[tiab] OR Labor, Obstetric[Mesh])** AND (**Data Collection[Mesh])** AND (**Population Surveillance[Mesh] OR** Surveillance[tiab] OR Information System*[tiab] OR Registry[tiab]) AND (Pregnancy[Mesh] OR Pregnan*[tiab] OR Maternal[tiab])

*Neonatal outcomes*

**Neonatal death (Early/Late)**

(Feasibility[tiab] OR Barrier*[tiab] OR Facilita*[tiab]) AND (**Perinatal Death[Mesh] OR Perinatal Death*[tiab] OR Neonatal Death*[tiab] OR Newborn Death*[tiab])** AND (**Data Collection[Mesh])** AND (**Population Surveillance[Mesh] OR** Surveillance[tiab] OR Information System*[tiab] OR Registry[tiab])

**Neonatal infection (symptomatic/ confirmed)**

(Feasibility[tiab] OR Barrier*[tiab] OR Facilita*[tiab]) AND (**Epidemics[Mesh] OR** Neonatal infection **AND Epidemic*[tiab] OR Pandemi*[tiab] OR Outbreak*[tiab])** AND (**Data Collection[Mesh])** AND (**Population Surveillance[Mesh] OR** Surveillance[tiab] OR Information System*[tiab] OR Registry[tiab]) AND (Infant, Newborn[Mesh] OR Newborn*[tiab] OR Neonate*[tiab])

**Severe disease**

(Feasibility[tiab] OR Barrier*[tiab] OR Facilita*[tiab]) AND (**Epidemics[Mesh] OR Epidemic*[tiab] OR Pandemi*[tiab] OR Outbreak*[tiab])** AND (**Data Collection[Mesh])** AND (**Population Surveillance[Mesh] OR** Surveillance[tiab] OR Information System*[tiab] OR Registry[tiab]) AND (**Infant, Newborn[Mesh] OR Newborn*[tiab] OR Neonate*[tiab])**

**Vertical transmission**

(Feasibility[tiab] OR Barrier*[tiab] OR Facilita*[tiab]) AND (**Infectious Disease Transmission, Vertical[Mesh] OR “Mother Child”[tiab:~2] OR “Fetomaternal Transmission”[tiab:~3] OR Maternal-Fetal[tiab] OR Vertical Transmission[tiab])** AND (**Population Surveillance[Mesh] OR** Surveillance[tiab] OR Information System*[tiab] OR Registry[tiab])

**Birth Asphyxia/ Need for neonatal resuscitation immediately after birth/Low Apgar score**

(Feasibility[tiab] OR Barrier*[tiab] OR Facilita*[tiab]) AND (**Asphyxia Neonatorum[Mesh] OR** Birth Asphyxia**[tiab] OR Neonate** Asphyxia**[tiab] OR Newborn** Asphyxia**[tiab] OR Resuscitation[Mesh] OR Resuscitation[tiab] OR Apgar Score[Mesh] OR Low Apgar[tiab])** AND (**Population Surveillance[Mesh] OR** Surveillance[tiab] OR Information System*[tiab] OR Registry[tiab]) AND (**Infant, Newborn[Mesh] OR Newborn*[tiab] OR Neonate*[tiab])**

**Neonatal sepsis**

(Feasibility[tiab] OR Barrier*[tiab] OR Facilita*[tiab]) AND (**Neonatal Sepsis[Mesh] OR “Neonatal Sepsis”[tiab:~3] OR “Neonate Sepsis”[tiab:~3] OR “Newborn Sepsis”[tiab:~3] OR Onset Sepsis[tiab])** AND (**Population Surveillance[Mesh] OR** Surveillance[tiab] OR Information System*[tiab] OR Registry[tiab]) AND (**Infant, Newborn[Mesh] OR Newborn*[tiab] OR Neonate*[tiab])**

**Respiratory distress (WHO)**

(Feasibility[tiab] OR Barrier*[tiab] OR Facilita*[tiab]) AND (**Respiratory Distress Syndrome, Newborn[Mesh] OR Respiratory Distress[tiab])** AND (**Population Surveillance[Mesh] OR** Surveillance[tiab] OR Information System*[tiab] OR Registry[tiab]) AND (**Infant, Newborn[Mesh] OR Newborn*[tiab] OR Neonate*[tiab])**

**Low birth weight**

(Feasibility[tiab] OR Barrier*[tiab] OR Facilita*[tiab]) AND (**Infant, Low Birth Weight[Mesh] OR Low-Birth-Weight[tiab] OR Low Birthweight[tiab] OR “Small Gestational Age”[tiab:~2] OR LBW[tiab] OR VLBW[tiab])** AND (**Population Surveillance[Mesh] OR** Surveillance[tiab] OR Information System*[tiab] OR Registry[tiab])

**Prematurity**

(Feasibility[tiab] OR Barrier*[tiab] OR Facilita*[tiab]) AND (**Premature Birth[Mesh] OR Premature[tiab] OR Preterm[tiab] OR Infant, Premature[Mesh])** AND (**Population Surveillance[Mesh] OR** Surveillance[tiab] OR Information System*[tiab] OR Registry[tiab])

**Neonatal admission to intensive care unit**

(Feasibility[tiab] OR Barrier*[tiab] OR Facilita*[tiab]) AND (**Intensive Care, Neonatal[Mesh] OR Intensive Care Units, Neonatal[Mesh] OR Neonatal ICU[tiab] OR Newborn ICU[tiab] OR Newborn Intensive-Care*[tiab] OR Neonate Intensive-Care*[tiab] OR NICU[tiab])** AND (**Population Surveillance[Mesh] OR** Surveillance[tiab] OR Information System*[tiab] OR Registry[tiab])

**Neonatal cardio-pulmonary resuscitation**

(Feasibility[tiab] OR Barrier*[tiab] OR Facilita*[tiab]) AND (**Resuscitation[Mesh] OR Resuscitation[tiab])** AND (**Population Surveillance[Mesh] OR** Surveillance[tiab] OR Information System*[tiab] OR Registry[tiab]) AND (**Infant, Newborn[Mesh] OR Newborn*[tiab] OR Neonate*[tiab])**

**Neonatal mechanical ventilation**

(Feasibility[tiab] OR Barrier*[tiab] OR Facilita*[tiab]) AND (**Respiration, Artificial[Mesh] OR Artificial Respiration[tiab] OR Mechanical Respiration[tiab] OR Artificial Ventilation[tiab] OR Mechanichal Ventilation[tiab])** AND (**Population Surveillance[Mesh] OR** Surveillance[tiab] OR Information System*[tiab] OR Registry[tiab]) AND (**Infant, Newborn[Mesh] OR Newborn*[tiab] OR Neonate*[tiab])**

**Any congenital anomalies**

(Feasibility[tiab] OR Barrier*[tiab] OR Facilita*[tiab]) AND (**Congenital Abnormalities[Mesh] OR Congenital Abnormal*[tiab] OR Deformit*[tiab] OR Malformation*[tiab] OR Fetal Anomal*[tiab])** AND (**Population Surveillance[Mesh] OR** Surveillance[tiab] OR Information System*[tiab] OR Registry[tiab]) AND (**Infant, Newborn[Mesh] OR Newborn*[tiab] OR Neonate*[tiab])**

**Gestational Age**

(Feasibility[tiab] OR Barrier*[tiab] OR Facilita*[tiab]) AND (**Gestational Age[Mesh] OR Gestational Age*[tiab] OR Fetal Age*[tiab] OR Fetal Maturity[tiab] OR Fetal Development[tiab])** AND (**Data Collection[Mesh])** AND (**Population Surveillance[Mesh] OR** Surveillance[tiab] OR Information System*[tiab] OR Registry[tiab])

**Birth weight**

(Feasibility[tiab] OR Barrier*[tiab] OR Facilita*[tiab]) AND (**Birth Weight[Mesh] OR Birth Weight[tiab] OR Birthweight[tiab] OR Gestational Age[tiab])** AND (**Population Surveillance[Mesh] OR** Surveillance[tiab] OR Information System*[tiab] OR Registry[tiab])

**Skin-to-skin**

(Feasibility[tiab] OR Barrier*[tiab] OR Facilita*[tiab]) AND (**Kangaroo-Mother Care Method[Mesh] OR Kangaroo Mother[tiab] OR Skin-to-Skin[tiab] OR KMC[tiab])** AND (**Population Surveillance[Mesh] OR** Surveillance[tiab] OR Information System*[tiab] OR Registry[tiab])

**Breastfeeding**

(Feasibility[tiab] OR Barrier*[tiab] OR Facilita*[tiab]) AND (**Breast Feeding[Mesh] OR Breast Feed*[tiab] OR Breastfeed*[tiab] OR Breast Fed[tiab] OR Breastfed[tiab])** AND (**Population Surveillance[Mesh] OR** Surveillance[tiab] OR Information System*[tiab] OR Registry[tiab])

# Supplementary File S4. Delphi Surveys Participants

Adejumoke Idowu Ayede

Albert Manasyan

Alberto Toso

Alejandra Gurtman

Alexandre Delamou

Amy Boldosser-Boesch

Andrew Stergachis

Ashraf Nabhan

Atf Gherissi

Azeem Walele

Babagana Bako

Barbara Rawlins

Boris Groisman

Brendan Carvalho

Camilla Pickles

Carolina Carvalho Ribeiro do Valle

Caroline Homer

Charles Christoph Roehr

Christina Ricci

Claire Thorne

Clare Whitehead

Cristiana Toscano

Cristina Cuesta-Zamora

Cyril Engmann

Courtney Carson

Dana Meaney-Delman

Daniele De Luca

Daniela Draghici

Daniela Noris Vásquez

David Kimberlin

David A. Schwartz

Deepak Chawla

Donna R. McCarraher

Doris Mollel

Dorotheah Obiri

Eleni Vavouraki

Elhadi Miskeen

Eliana Marengo

Ellen O'Keeffe

Erika Ota

Fatema Ashraf

Fleda Mask Jackson

Flor Munoz

Fook-Choe Cheah

France Donnay

Gabriela Tavchioska

Ghislain Franck Houndjahoue

Glen Mola

Gloria Carmona

Guilherme Amaral Calvet

Hama Diallo

Hellen Barsosio

Hellen Mutsi

Hemantha S enanayake

Henri Gautier Ouedraogo

İlknur Okay

Isabel Chenai Chipunza

Jackeline Alger

Jean Paul Ndayizeye

Jonathan Luke Richardson

José Guilherme Cecatti

José Antonio Rojas-Suarez

Joyce L. Browne

Julián Gustavo Antman

Kara Blackburn

Katharina Hartmann

Kaveri Mayra

Ken Takahashi

Koiwah Koi-Larbi Ofosuapea

Kylie Pussell

Laura Lamberti

Laura Yates

Leah Greenspan

Léo Pomar

Lina Bergman

Loïc Sentilhes

Lola Madrid

Luigi Gagliardi

Lynne M. Mofenson

Magdalena Lyimo

Manal Younus

Marge Berer

María Aurelia Giboin Mazzola

Maria Margarita Lota

María Fernanda Escobar Vidarte

María José Rodríguez-Sibaja

Maria Laura Costa do Nascimento

Marian Knight

Marleen van Gelder

Marynéa Silva do Vale

Meghan B. Azad

Michael G. Gravett

Michal Lipschuetz

Michelle Sadler

Mirna Montenegro Rangel

Nadia A. Sam-Agudu

Nasim Akhtar

Nasrin Changizi

Nicole Lurie

Nihaya Al-sheyab

Niveen M. E. Abu-Rmeileh

Noreen Zafar

Nzelle Delphine Kayem

Olarik Musigavong

Oleksandra Balyasna

Olufunke Bolaji

Paolo Ivo Cavoretto

Paolo Manzoni

Petr Velebil

Phoebe C.M. Williams

Pierre Buekens

Pius OKong

Priya Soma-Pillay

Raanan Raz

Ramon Escuriet

Redeat Workneh

Rhoda Amafumba

Ricardo Nieto

Rose Bisoborwa Mukisa

Rosemary Njura Njogu

Rosnah Sutan

Sangappa Dhaded

Safa Elhassan

Sarah Jorgensen

Satoru Ikenoue

Selma Hajri

Serge Alain Tougma

Shabina Ariff

Shivaprasad S. Goudar

Shuby Puthussery

Silke Mader

Soledad Puppo

Sonia Deantoni

Soo Downe

Stelian Hodorogea

Stephanie Dellicour

Stephanie Pabón Lozano

Tavitiya Sudjaritruk

Therese Mpiempie Ngamasata Maleluka

Thomas F. McElrath

Tippawan Liabsuetrakul

Uduak Okomo

Ursula Winterfeld

Walusa Assad Gonçalves-Ferri

Wendy E. Pollock

Yousef Saleh Khader

Yuefang Huang

Zaleha Abdullah Mahdy

# Supplementary File S5. Consensus meetings list of participants

**DEPARTMENT OF SEXUAL AND REPRODUCTIVE HEALTH AND RESEARCH**

**WHO CONSULTATION ON THE DEVELOPMENT OF A CORE OUTCOME SET (COS) FOR MATERNAL AND PERINATAL HEALTH RESEARCH AND SURVEILLANCE**

**IN THE CONTEXT OF EMERGING AND ONGOING EPIDEMIC THREATS**

14-15 February 2024

**List of Participants**

**Temporary Advisers**

**Jackeline ALGER**

Department of Clinical Laboratory

University Hospital

Tegucigalpa

Honduras

**Hellen BARSOSIO**

Maternal and Newborn Health Research

Kenya Medical Research Institute (KEMRI)

Nairobi

Kenya

**Kara BLACKBURN**

Burnet Institute

Melbourne

Australia

**Olufunke BOLAJI**

African Neonatal Association

Ido Ekiti Ekiti

Nigeria

**Courtney CARSON (unable to attend)**

Pandemic Action Network

New York

USA

**Daniele DE LUCA**

APHP-Paris Saclay University

“A. Béclère” Medical Center

Paris

France

**Sangappa DHADED**

KLE Academy of Higher Education and Research

Karnataka

India

**Cyril ENGMANN**

PATH

Seattle

USA

**Maria Fernanda ESCOBAR VIDARTE**

Global Health Equity Unit

Valle del Lili Foundation

Cali

Colombia

**Ramón ESCURIET**

[National](https://www.birmingham.ac.uk/research/who-collaborating-centre/index.aspx) Health Service

Barcelona

Spain

**Marian KNIGHT**

National Perinatal Epidemiology Unit

Nuffield Department of Population Health

University of Oxford

Oxford

UK

**Maria Margarita LOTA**

Department of Medical Microbiology College of Public Health

University of the Philippines

Manila

Philippines

**Silke MADER**

European Foundation for the Care of Newborn Infants (EFONI)

Munich

Germany

**Lola MADRID CASTILLO**

Department of Infectious Disease Epidemiology and International Health

London School of Hygiene and Tropical Medicine (LSHTM)

London

UK

**Mirna MONTENEGRO RANGEL**

Observatory for Sexual and Reproductive Health (OSAR)

Guatemala City

Guatemala

**Rose MUKISA-BISOBORWA**

White Ribbon Alliance Uganda

Kampala

Uganda

**Flor MUNOZ-RIVAS**

Pediatrics-Infectious Disease

Baylor College of Medicine

Houston, TX

USA

**Uduak OKOMO**

Medical Research Council (MRC) Unit

London School of Hygiene and Tropical Medicine (LSHTM)

Banjul

The Gambia

**Pius OKONG**

Uganda Martyrs University (UMU)

Nkozi

Uganda

**David SCHWARTZ**

Perinatal Pathology Consulting

Atlanta, GA

USA

**Tavitiya SUDJARITRUK**

Department of Pediatrics

Chiang Mai University

Chiang Mai

Thailand

**Laura YATES**

University of KwaaZulu-Natal

Durban

South Africa

**Manal YOUNUS**

Iraqi Pharmacovigilance Center

Ministry of Health

Baghdad

Iraq

**Noreen ZAFAR**

Girls and Women's Health Initiative

Lahore

Pakistan

**Institute for Clinical Effectiveness and Health Policy (IECS)**

**Juan Pedro ALONSO**

IECSBuenos Aires

Argentina

Email: [jalonso@iecs.org.ar](mailto:jalonso@iecs.org.ar)

**Maria BELIZAN**

IECS

Buenos Aires

Argentina

Email: [mbelizan@iecs.org.ar](mailto:mbelizan@iecs.org.ar)

**Mabel BERRUETA**

IECS

Buenos Aires

Argentina

Email: [mberrueta@iecs.org.ar](mailto:mberrueta@iecs.org.ar)

**Karen KLEIN**

IECS

Buenos Aires

Argentina

Email: [kklein@iecs.org.ar](mailto:kklein@iecs.org.ar)

**Veronica PINGRAY**

IECS

Buenos Aires

Argentina

Email: [vpingray@iecs.org.ar](mailto:vpingray@iecs.org.ar)

**WHO Secretariat**

**Magdalena BABINSKA**

Maternal and Perinatal Health

Department of Sexual and Reproductive

Health and Research

Email: [babinskam@who.int](mailto:babinskam@who.int)

**Mercedes BONET**

Maternal and Perinatal Health

Department of Sexual and Reproductive

Health and Research

Email: [bonetm@who.int](mailto:bonetm@who.int)

**Janet DIAZ (unable to attend)**

Health Care Readiness WHE

Email: [diazj@who.int](mailto:diazj@who.int)

**Edna KARA**

Sexual Health and Wellbeing Across the Life Course

Department of Sexual and Reproductive

Health and Research

Email: [karae@who.int](mailto:karae@who.int)

**Caron Rahn KIM**

Prevention of Unsafe Abortion

Department of Sexual and Reproductive

Health and Research

Email: [kimca@who.int](mailto:kimca@who.int)

**Smaragda LAMPRIANOU**

Access to Medicines and Health Products

Email: [lamprianous@who.int](mailto:lamprianous@who.int)

**Olufemi OLADAPO**

Maternal and Perinatal Health

Department of Sexual and Reproductive

Health and Research

Email: [oladapoo@who.int](mailto:oladapoo@who.int%20)

**Soe Soe THWIN** (unable to attend)

Research Leadership and Knowledge Management

Department of Sexual and Reproductive

Health and Research

Email: [thwins@who.int](mailto:thwins@who.int)

# Table S1. Additional outcomes suggested by participants

| **Domain** | **Outcome** |
| --- | --- |
| Functioning - Delivery of care | Maternal satisfaction (with care received) |
|  | Rooming in |
|  | Companionship during childbirth |
| Functioning - Delivery of care - Preventive and Therapeutic intervention related to outbreak | Prophylactic steroids during pregnancy |
|  | Severe, life-threatening, disabling or death adverse event |
|  | Severe, life-threatening, disabling or death medical error |
|  | Gestational age at medication use end |
|  | Maternal drug therapy |
| Functioning - Delivery of care - Obstetric management | Obstetrical anesthesia complications |
| Functioning - Delivery of care - Clinical Management | Patient readmission |
| Functioning - Emotional functioning/well- being | Sexual satisfaction |
| Physiological/clinical - Infection and infestation outcomes | Sexually transmitted infections during pregnancy |
| Physiological/clinical - Maternal Morbidity not related to pregnancy (by system) | Maternal Near Miss |
| Physiological/clinical - Other | Use of non-prescribed drugs to prevent infection |
| Mortality / vital status | Cause of death |
|  | Histopathological studies performed (biopsy / autopsy) |
| Physiological/clinical - Neonatal morbidity | Neonatal near miss |
|  | Neurodevelopment outcomes |
|  | Recurrence of neonatal morbidity |
| Physiological/clinical-Infection and infestation outcomes | Neonatal vaccination |
|  | Neonatal drug therapy |

# Table S2. Results from Round 1 and 2. Maternal outcomes

| **Outcome** | **Round 1** | | | | | **Round 2** | | | | |  |
| --- | --- | --- | --- | --- | --- | --- | --- | --- | --- | --- | --- |
|  | Maternal health | Neonatal health | Public health | Civil society |  | Maternal health | Neonatal health | Public health | Civil society |  |  |
|  | n=73 | n=30 | n=26 | n=21 |  | n=69 | n=29 | n=23 | n=18 |  |  |
|  | n (%) | n (%) | n (%) | n (%) | Status | n (%) | n (%) | n (%) | n (%) | Status |  |
| **Maternal and perinatal mortality / vital status** |  |  |  |  |  |  |  |  |  |  |  |
| Live birth | 66 (92) | 26 (87) | 24 (92) | 17 (81) | Agree to include | NA | NA | NA | NA | NA |  |
| Maternal death | 70 (97) | 30 (100) | 26 (100) | 20 (95) | Agree to include | NA | NA | NA | NA | NA |  |
| Perinatal death | 68 (96) | 30 (100) | 26 (100) | 19 (90) | Agree to include | NA | NA | NA | NA | NA |  |
| Stillbirth | 69 (96) | 30 (100) | 25 (96) | 18 (86) | Agree to include | NA | NA | NA | NA | NA |  |
| **Physiological/clinical - Maternal Morbidity** |  |  |  |  |  |  |  |  |  |  |  |
| Ectopic/molar pregnancy | 27 (39) | 8 (30) | 8 (35) | 7 (47) | Agree to exclude | NA | NA | NA | NA | NA |  |
| Hyperemesis gravidarum | 11 (17) | 4 (14) | 2 (10) | 3 (9) | Agree to exclude | NA | NA | NA | NA | NA |  |
| Insufficient cervix | 17 (25) | 8 (29) | 4 (17) | 7 (41) | Agree to exclude | NA | NA | NA | NA | NA |  |
| Intrahepatic cholestasis of pregnancy | 15 (24) | 9 (32) | 4 (20) | 3 (27) | Agree to exclude | NA | NA | NA | NA | NA |  |
| Oligohydramnios | 24 (38) | 14 (47) | 7 (32) | 5 (47) | Agree to exclude | NA | NA | NA | NA | NA |  |
| Polyhydramnios | 19 (31) | 14 (47) | 5 (24) | 4 (33) | Agree to exclude | NA | NA | NA | NA | NA |  |
| Diseases of the urinary system | 16 (24) | 6 (21) | 4 (18) | 6 (38) | Agree to exclude | NA | NA | NA | NA | NA |  |
| Disorders of the thyroid gland or thyroid hormones system | 15 (22) | 8 (29) | 2 (10) | 7 (41) | Agree to exclude | NA | NA | NA | NA | NA |  |
| Haematologic disorders | 33 (50) | 13 (45) | 8 (38) | 7 (44) | Agree to exclude | NA | NA | NA | NA | NA |  |
| Liver disorders | 24 (36) | 8 (29) | 7 (35) | 7 (44) | Agree to exclude | NA | NA | NA | NA | NA |  |
| Neurologic disorders | 26 (38) | 11 (41) | 6 (30) | 7 (44) | Agree to exclude | NA | NA | NA | NA | NA |  |
| Peritoneal effusion | 11 (18) | 8 (30) | 6 (29) | 4 (31) | Agree to exclude | NA | NA | NA | NA | NA |  |
| Postpartum haemorrhage | 59 (87) | 27 (90) | 20 (84) | 19 (95) | Agree to include | NA | NA | NA | NA | NA |  |
| Antenatal bleeding (unspecified etiology) | 30 (43) | 20 (67) | 9 (38) | 11 (19) | No agreement | 37 (55) | 18 (62) | 6 (27) | 11 (73) | No agreement |  |
| Chorioamnionitis | 40 (61) | 23 (77) | 10 (45) | 6 (50) | No agreement | 51 (76) | 21 (72) | 13 (59) | 10 (77) | No agreement |  |
| Gestational diabetes mellitus | 40 (56) | 22 (73) | 10 (42) | 8 (53) | No agreement | 43 (63) | 21 (72) | 13 (57) | 11 (69) | No agreement |  |
| Hypertensive disorders of pregnancy | 55 (77) | 27 (90) | 16 (67) | 13 (76) | No agreement | 63 (93) | 26 (90) | 18 (78) | 15 (94) | No agreement* |  |
| Morbidly adherent placenta | 30 (48) | 14 (48) | 9 (40·91) | 8 (57) | Agree to exclude | NA | NA | NA | NA | NA |  |
| Amniotic fluid embolism | 37 (58) | 13 (48) | 12 (55) | 9 (29) | Agree to exclude | NA | NA | NA | NA | NA |  |
| Placenta previa | 33 (49) | 17 (57) | 11 (48) | 11 (65) | Agree to exclude | NA | NA | NA | NA | NA |  |
| Placental abruption | 43 (65) | 23 (77) | 14 (61) | 11 (69) | No agreement | 52 (80·00) | 24 (83) | 14 (64) | 15 (94) | No agreement |  |
| Premature rupture of membranes | 38 (56) | 24 (80) | 14 (56) | 11 (61) | No agreement | 43 (65) | 23 (79) | 10 (43) | 11 (65) | No agreement |  |
| Preterm birth / delivery (unspecify aetiology) | 61 (86) | 31 (100) | 21 (84) | 15 (75) | No agreement | 65 (96) | 29 (100) | 21 (91) | 14 (88) | Agree to include |  |
| Preterm birth/ delivery spontaneous | 61 (86) | 31 (100) | 19 (76) | 16 (80) | No agreement | 66 (97) | 29 (100) | 20 (87) | 16 (100) | Agree to include |  |
| Preterm labour | 52 (74) | 25 (83) | 16 (64) | 12 (63) | No agreement | 58 (88) | 27 (93) | 14 (61) | 13 (76) | No agreement |  |
| Spontaneus abortion | 47 (67) | 22 (76) | 17 (71) | 12 (63) | No agreement | 47 (71) | 21 (75) | 18 (78) | 11 (65) | No agreement |  |
| Threatened abortion | 28 (40) | 16 (57) | 9 (38) | 10 (56) | Agree to exclude | NA | NA | NA | NA | NA |  |
| Uterine rupture | 45 (66) | 21 (72) | 17 (74) | 15 (79) | No agreement | 50 (75) | 17 (61) | 18 (86) | 15 (94) | No agreement |  |
| Acute renal injury | 36 (53) | 16 (57) | 8 (36) | 10 (63) | Agree to exclude | NA | NA | NA | NA | NA |  |
| Any respiratory disorders (other than pneumonia) | 30 (44) | 12 (41) | 11 (55) | 6 (38) | Agree to exclude | NA | NA | NA | NA | NA |  |
| Shock | 53 (76) | 23 (77) | 18 (78) | 12 (71) | No agreement | 62 (94) | 23 (79) | 21 (95) | 12 (80) | No agreement |  |
| Thrombo-embolic event | 49 (71) | 23 (77) | 20 (87) | 11 (67) | No agreement | 57 (86) | 19 (68) | 19 (90) | 11 (79) | No agreement* |  |
| **Physiological/clinical - Maternal Morbidity not related to pregnancy** |  |  |  |  |  |  |  |  |  |  |  |
| **Physiological/clinical - Placenta related outcomes** |  |  |  |  |  |  |  |  |  |  |  |
| Placental or umbilical cord conditions | 25 (37) | 17 (61) | 7 (32) | 8 (50) | Agree to exclude | NA | NA | NA | NA | NA |  |
| Villitis/intervillositis | 19 (31) | 13 (50) | 5 (26) | 0 (0) | Agree to exclude | NA | NA | NA | NA | NA |  |
| **Physiological/clinical - Maternal infection and infestation outcomes** |  |  |  |  |  |  |  |  |  |  |  |
| Abnormal imaging findings | 21 (32) | 12 (43) | 7 (32) | 7 (44) | Agree to exclude | NA | NA | NA | NA | NA |  |
| Endometritis | 34 (48) | 13 (46) | 8 (35) | 4 (25·00) | Agree to exclude | NA | NA | NA | NA | NA |  |
| Vertical transmission | 59 (84) | 26 (90) | 22 (88) | 13 (93) | Agree to include | NA | NA | NA | NA | NA |  |
| Gestational age at infection | 45 (65) | 19 (66) | 13 (52) | 8 (47) | Agree to exclude | NA | NA | NA | NA | NA |  |
| Maternal asymptomatic infection (related outbreak disease) | 42 (59) | 18 (64) | 15 (60) | 9 (50) | Agree to exclude | NA | NA | NA | NA | NA |  |
| Maternal confirmed infection (related outbreak disease) | 49 (69) | 26 (90) | 19 (76) | 12 (67) | No agreement | 59 (86·76) | 28 (96·55) | 22 (95·65) | 14 (82·35) | Agree to include |  |
| Maternal symptomatic infection (related outbreak disease) | 54 (75) | 26 (90) | 20 (80) | 11 (61) | No agreement | 63 (93) | 26 (93) | 21 (92) | 13 (76) | No agreement* |  |
| Nosocomial infection | 36 (51) | 16 (57) | 15 (60) | 8 (50) | Agree to exclude | NA | NA | NA | NA | NA |  |
| Pneumonia | 98 (69) | 47 (81) | 25 (51) | 23 (66) | No agreement | 48 (71) | 24 (83) | 11 (52) | 12 (71) | No agreement |  |
| Progression to severe/critical disease (related outbreak disease) | 64 (89) | 27 (96) | 20 (83) | 13 (76) | No agreement | 65 (96) | 28 (100) | 23 (100) | 16 (94) | Agree to include |  |
| Protective antibodies | 30 (43) | 15 (54) | 12 (48) | 7 (47) | Agree to exclude | NA | NA | NA | NA | NA |  |
| Recovered | 39 (56) | 17 (63) | 15 (63) | 9 (60) | Agree to exclude | NA | NA | NA | NA | NA |  |
| Sepsis | 64 (90) | 25 (86) | 23 (88) | 13 (68) | No agreement | 68 (100) | 26 (90) | 23 (100) | 14 (88) | Agree to include |  |
| Seroconversion | 41(61) | 15 (54) | 14 (58) | 6 (46) | Agree to exclude | NA | NA | NA | NA | NA |  |
| **Physiological/clinical - Labour and delivery characteristics** |  |  |  |  |  |  |  |  |  |  |  |
| Complications of labour or delivery | 56 (81) | 23 (82) | 18 (72) | 13 (72) | No agreement | 51 (75) | 20 (77) | 17 (77) | 14 (88) | No agreement |  |
| Complications of third stage of labour | 51 (74) | 21 (75) | 13 (54) | 14 (74) | No agreement | 48 (71) | 18 (64) | 12 (60) | 12 (60) | No agreement |  |
| Gestational age at delivery | 60 (86) | 27 (96) | 17 (65) | 13 (65) | No agreement | 64 (94) | 28 (97) | 19 (83) | 19 (83) | Agree to include |  |
| Labour onset | 33 (47) | 17 (61) | 8 (32) | 7 (41) | Agree to exclude | NA | NA | NA | NA | NA |  |
| Mode of delivery | 47 (67) | 20 (69) | 12 (46) | 12 (63) | No agreement | 50 (74) | 21 (72) | 13 (57) | 13 (76) | No agreement |  |
| **Physiological/clinical - Fetal outcomes** |  |  |  |  |  |  |  |  |  |  |  |
| Decreased fetal movements | 39 (55) | 17 (57) | 13 (54) | 12 (71) | No agreement | 39 (57) | 19 (66) | 9 (41) | 12 (80) | No agreement |  |
| Foetal growth restriction | 60 (85) | 27 (90) | 16 (67) | 12 (67) | No agreement | 63 (93) | 28 (97) | 15 (71) | 12 (75) | No agreement* |  |
| Hydrops fetalis | 36 (59) | 24 (80) | 11 (52) | 4 (40) | No agreement | 34 (58) | 21 (72) | 8 (44) | 7 (70) | No agreement |  |
| Non-reassuring fetal status | 35 (55) | 23 (77) | 15 (63) | 9 (69) | No agreement | 32 (50) | 17 (63) | 7 (37) | 10 (83) | No agreement |  |
| Multiple gestation | 39 (56) | 19 (63) | 24 (54) | 10 (59) | Agree to exclude | NA | NA | NA | NA | NA |  |
| **Maternal social functioning** |  |  |  |  |  |  |  |  |  |  |  |
| Mother-newborn isolation | 36 (50) | 19 (63) | 9 (38) | 13 (62) | Agree to exclude | NA | NA | NA | NA | NA |  |
| Social support | 37 (51) | 19 (63) | 11 (42) | 16 (76) | No agreement | 33 (48) | 17 (59) | 10 (43) | 14 (78) | No agreement |  |
| Violence | 50 (69) | 19 (63) | 16 (66) | 17 (81) | No agreement | 48 (71) | 20 (69) | 16 (70) | 15 (83) | No agreement |  |
| Worries | 25 (35) | 11 (37) | 7 (28) | 9 (45) | Agree to exclude | NA | NA | NA | NA | NA |  |
| **Maternal emotional functioning/well-being** |  |  |  |  |  |  |  |  |  |  |  |
| Insomnia | 25 (35) | 11 (37) | 4 (16) | 8 (38) | Agree to exclude | NA | NA | NA | NA | NA |  |
| Panic | 27 (38 | 15 (50) | 5 (21) | 9 (43) | Agree to exclude | NA | NA | NA | NA | NA |  |
| Resiliance | 27 (39) | 11 (39) | 5 (21) | 10 (50) | Agree to exclude | NA | NA | NA | NA | NA |  |
| Anxiety | 39 (55) | 16 (53) | 9 (36) | 11 (52) | Agree to exclude | NA | NA | NA | NA | NA |  |
| Depression | 52 (73) | 22 (73) | 12 (48) | 15 (71) | No agreement | 45 (65) | 14 (48) | 9 (39) | 12 (67) | No agreement |  |
| Peripartum psychiatric disorder (unspecified) | 45 (65) | 20 (67) | 12 (50) | 15 (75) | No agreement | 41 (59) | 15 (52) | 10 (45) | 11 (65) | No agreement |  |
| Psychological well-being | 39 (55) | 17 (57) | 7 (29) | 15 (71) | No agreement | 34 (49) | 16 (55) | 6 (27) | 10 (56) | No agreement |  |
| Stress | 31 (44) | 12 (41) | 6 (25) | 13 (62) | Agree to exclude | NA | NA | NA | NA | NA |  |
| **Maternal functioning - Delivery of care** |  |  |  |  |  |  |  |  |  |  |  |
| Maternal admission to intensive care unit | 65 (93) | 25 (86) | 24 (92) | 16 (84) | Agree to include | NA | NA | NA | NA | NA |  |
| Blood transfusion | 103 (74) | 39 (66) | 36 (69) | 27 (71) | No agreement | 48 (73) | 20 (74) | 17 (74) | 11 (73) | No agreement |  |
| Maternal hospital admission/hospital stay | 46 (66) | 19 (66) | 15 (58) | 12 (67) | No agreement | 45 (67) | 19 (70) | 14 (61) | 10 (67) | No agreement |  |
| Maternal mechanical ventilation | 60 (86) | 25 (86) | 24 (92) | 15 (79) | No agreement | 60 (90) | 24 (83) | 21 (91) | 15 (94) | Agree to include |  |
| Maternal oxygen support | 51 (73) | 26 (90) | 22 (85) | 16 (85) | No agreement | 54 (81) | 23 (79) | 21 (91) | 14 (88) | No agreement |  |
| Augmentation of labour | 26 (38) | 8 (28) | 9 (38) | 6 (35) | Agree to exclude | NA | NA | NA | NA | NA |  |
| Episiotomy | 22 (32) | 2 (7) | 5 (21) | 4 (24) | Agree to exclude | NA | NA | NA | NA | NA |  |
| Hysterectomy | 49 (71) | 16 (55) | 11 (46) | 9 (56) | No agreement | 42 (64) | 8 (29) | 5 (25) | 9 (69) | No agreement |  |
| Induced abortion | 43 (62) | 16 (57) | 16 (64) | 10 (56) | Agree to exclude | NA | NA | NA | NA | NA |  |
| Preterm birth/delivery provider initiated (induced or iatrogenic) | 58 (82) | 22 (76) | 18 (69) | 8 (47) | No agreement | 59 (86) | 26 (90) | 16 (70) | 14 (88) | No agreement |  |
| Screening for detection of aneuploidies | 19 (30) | 11 (39) | 6 (27) | 3 (25) | Agree to exclude | NA | NA | NA | NA | NA |  |
| **Maternal functioning - Cognitive functioning** | |  |  |  |  |  |  |  |  |  |  |
| Self-efficacy | 33 (49) | 11 (38) | 6 (25) | 11 (52) | Agree to exclude | NA | NA | NA | NA | NA |  |
| **Maternal Functioning - Preventive and therapeutic intervention related to outbreak** | | | |  |  |  |  |  |  |  |  |
| Gestational age at vaccination | 42 (61) | 26 (87) | 16 (62) | 11 (58) | No agreement | 46 (69) | 25 (86) | 17 (74) | 13 (81) | No agreement |  |
| Maternal immunization | 52 (73) | 24 (80) | 20 (77) | 15 (79) | No agreement | 58 (85) | 26 (90) | 18 (78) | 15 (83) | No agreement |  |
| Women views on vaccine acceptability | 38 (53·52) | 18 (62·07) | 11 (42·31) | 11 (55·00) | Agree to exclude | NA | NA | NA | NA | NA |  |
|  |  |  |  |  |  |  |  |  |  |  |  |
|  |  |  |  |  |  |  |  |  |  |  |  |
| Note: With variations depending on the outcome, on average, 7·7% of the participants in Round 1 and 0·55% in Round 2 abstained from voting because they perceived themselves as unable to rate the outcome due to a lack of expertise· | | | | | | |  |  |  |  |  |
| * These outcomes were deemed borderline if, in the second survey, ≥90% of participants in at least one stakeholder panel rated the outcome as critically important, did not have substantial overlap with included outcomes, were included within domains with substantial gaps, and were relevant to smaller panels· | | | | | | | | | | |  |

# Table S3· Results from Round 1 and 2· Neonatal outcomes

| **Outcome** | **Round 1** | | | | | **Round 2** | | | | | | | |  |
| --- | --- | --- | --- | --- | --- | --- | --- | --- | --- | --- | --- | --- | --- | --- |
|  | Maternal health | Neonatal health | Public health | Civil society |  | Maternal health | Neonatal health | Public health | | Civil society | |  | |  |
|  | n=73 | n=30 | n=26 | n=21 |  | n=69 | n=29 | n=23 | | n=18 | |  | |  |
|  | n (%) | n (%) | n (%) | n (%) | Status | n (%) | n (%) | n (%) | | n (%) | | Status | |  |
| **Neonatal Mortality** |  |  |  |  |  |  |  |  | |  | |  | |  |
| Neonatal death | 72 (100) | 30 (100) | 25 (96) | 19 (95) | Agree to include | NA | NA | NA | | NA | | NA | |  |
| **Physiological/clinical - Neonatal outcomes** |  |  |  |  |  |  |  |  | |  | |  | |  |
| Birth weight | 61 (84) | 28 (93) | 19 (73) | 14 (70) | No agreement | 63 (91) | 29 (100) | 19 (83) | | 14 (82) | | Agree to include | |  |
| Breastfeeding at first hour | 38 (54) | 15 (50) | 10 (38) | 13 (65) | Agree to exclude | NA | NA | NA | | NA | | NA | |  |
| Gestational age at birth | 66 (90) | 30 (100) | 20 (77) | 16 (80) | No agreement | 66 (96) | 29 (100) | 19 (83) | | 16 (94) | | Agree to include | |  |
| Birth weight for gestational age | 59 (81) | 25 (83) | 19 (73) | 14 (70) | No agreement | 62 (90) | 27 (93) | 15 (65) | | 15 (88) | | No agreement | |  |
| Type of feeding | 41 (57) | 18 (60) | 7 (27) | 9 (45) | Agree to exclude | NA | NA | NA | | NA | | NA | |  |
| **Physiological/clinical - Neonatal morbidity** |  |  |  |  |  |  |  |  | |  | |  | |  |
| Any cardiovascular disorder(s) | 46 (67) | 22 (73) | 12 (50) | 10 (63) | No agreement | 48 (71) | 20 (71) | 12 (57) | | 12 (80) | | No agreement | |  |
| Any congenital malformation(s) or birth defects (non specified) | 52 (75) | 26 (87) | 14 (56) | 12 (71) | No agreement | 53 (78) | 25 (86) | 15 (68) | | 10 (71) | | No agreement | |  |
| Any Hematologic disorder(s) | 38 (55) | 23 (77) | 11 (46) | 10 (67) | No agreement | 39 (57) | 17 (59) | 7 (35) | | 9 (75) | | No agreement | |  |
| Neurologic disorder(s) | 44 (63) | 22 (73) | 13 (54) | 10 (62) | No agreement | 49 (71) | 22 (76) | 11 (52) | | 10 (71) | | No agreement | |  |
| Any respiratory disorder(s) | 47 (68) | 24 (80) | 15 (63) | 11 (69) | No agreement | 55 (81) | 27 (93) | 15 (71) | | 12 (80) | | No agreement* | |  |
| APGAR score | 52 (72) | 21 (72) | 16 (64) | 13 (76) | No agreement | 56 (81) | 23 (82) | 15 (652) | | 13 (87) | | No agreement | |  |
| Arterial pH < 7;0 | 39 (60) | 22 (76) | 12 (55) | 11 (85) | No agreement | 39 (62) | 20 (71) | 10 (48) | | 10 (91) | | No agreement | |  |
| Birth Asphixia | 58 (82) | 29 (97) | 17 (71) | 15 (83) | No agreement | 65 (96) | 28 (97) | 18 (86) | | 14 (93) | | Agree to include | |  |
| Congenital malformations and deformations of the musculoskeletal system | 32 (47) | 25 (86) | 11 (46) | 9 (56) | No agreement | 29 (43) | 24 (83) | 11 (52) | | 7 (50) | | No agreement | |  |
| Congenital malformations of eye; ear; face and neck | 33 (49) | 25 (86) | 11 (46) | 10 (59 | No agreement | 27 (40) | 24 (83) | 10 (48 | | 6 (46) | | No agreement | |  |
| Congenital malformations of nervous system | 38 (55) | 27 (93) | 14 (58) | 10 (59) | No agreement | 39 (57) | 26 (90) | 13 (62) | | 9 (64) | | No agreement | |  |
| Congenital malformations of the circulatory system | 40 (57) | 26 (90) | 14 (58 | 11 (65) | No agreement | 37 (54) | 25 (89) | 15 (71) | | 9 (75) | | No agreement | |  |
| Congenital malformations of the genital organs and the urinary system | 36 (51) | 27 (93) | 12 (50) | 10 (59) | No agreement | 27 (40) | 26 (90) | 11 (52) | | 8 (57) | | No agreement | |  |
| Low or abnormal APGAR score | 47 (66) | 24 (80) | 12 (48) | 13 (81) | No agreement | 43 (63) | 20 (69) | 13 (59) | | 14 (93) | | No agreement | |  |
| Multiple malformations | 42 (60) | 27 (90) | 13 (52) | 11 (69) | No agreement | 45 (67) | 25 (86) | 14 (64) | | 13 (81) | | No agreement | |  |
| Meconium aspiration | 37 (53) | 19 (63) | 8 (33) | 7 (50) | Agree to exclude | NA | NA | NA | | NA | | NA | |  |
| Necrotizing enterocolitis | 45 (65) | 25 (83) | 11 (48) | 8 (62) | No agreement | 46 (71) | 20 (69) | 10 (48) | | 10 (48) | | No agreement | |  |
| Neonatal encephalopathy | 48 (68) | 27 (90) | 13 (54) | 8 (57) | No agreement | 56 (82) | 26 (90) | 14 (64) | | 8 (62) | | No agreement | |  |
| Neonatal hypoglycemia | 42 (60) | 24 (83) | 11 (48) | 10 (71) | No agreement | 48 (72) | 23 (82) | 12 (57) | | 12 (80) | | No agreement | |  |
| Neonatal hypothermia | 46 (65) | 22 (73) | 10 (43) | 10 (71) | No agreement | 46 (69) | 23 (79) | 12 (57) | | 13 (81) | | No agreement | |  |
| Neonatal intracranial hemorrhage | 54 (77) | 26 (87) | 17 (68) | 11 (79) | No agreement | 63 (94) | 25 (86) | 17 (77) | | 10 (77) | | No agreement | |  |
| Neonatal jaundice | 42 (59) | 15 (50) | 9 (38) | 9 (56) | Agree to exclude | NA | NA | NA | | NA | | NA | |  |
| Neonatal sepsis | 62 (87) | 26 (90) | 18 (72) | 13 (81) | No agreement | 67 (99) | 27 (93) | 19 (86) | | 13 (87) | | Agree to include | |  |
| Pneumonia | 98 (79) | 47 (81) | 25 (51) | 23 (65) | No agreement | 58 (85) | 23 (79) | 11 (50) | | 11 (79) | | No agreement | |  |
| Pulmonary hypertension | 39 (56) | 22 (76) | 10 (43) | 12 (80) | No agreement | 38 (57) | 21 (72) | 8 (40) | | 11 (79) | | No agreement | |  |
| Respiratory distress syndrome | 52 (75) | 28 (93) | 17 (67) | 11 (73) | No agreement | 61 (92) | 26 (90) | 18 (82) | | 12 (80) | | Agree to include | |  |
| Transient tachypnea | 28 (44) | 11 (37) | 9 (41) | 6 (50) | Agree to exclude | NA | NA | NA | | NA | | NA | |  |
| Weight loss | 28 (41) | 14 (47) | 11 (44) | 7 (45) | Agree to exclude | NA | NA | NA | | NA | | NA | |  |
| **Physiological/clinical - Neonatal infection and infestation outcomes** |  |  |  |  |  |  |  |  | |  | |  | |  |
| Neonatal asymptomatic infection (related outbreak disease) | 43 (62) | 20 (67) | 15 (58) | 10 (56) | No agreement | 46 (69) | 20 (69) | 15 (68) | | 12 (80) | | No agreement | |  |
| Neonatal confirmed infection (related outbreak disease) | 57 (83) | 28 (93) | 21 (81) | 13 (72) | No agreement | 64 (94) | 29 (100) | 22 (96) | | 15 (88) | | Agree to include | |  |
| Neonatal symptomatic infection (related outbreak disease) | 58 (83) | 29 (97) | 22 (85) | 13 (72) | No agreement | 67 (99) | 28 (97) | 22 (96) | | 15 (88) | | Agree to include | |  |
| Severe disease (related outbreak disease) | 63 (90) | 30 (100) | 22 (85) | 17 (89) | Agree to include | NA | NA | NA | | NA | | Agree to include | |  |
| **Neonatal Functioning-Deliver of care** |  |  |  |  |  |  |  |  | |  | |  | |  |
| Blood transfusion | 103 (74) | 39 (66) | 36 (69) | 27 (71) | No agreement | 49 (73) | 18 (62) | 13 (59) | | 12 (80) | | No agreement | |  |
| Cardipulmonary resuscitation | 62 (87) | 26 (87) | 19 (73· | 14 (82) | No agreement | 65 (96) | 24 (83) | 21 (91) | | 14 (93) | | Agree to include | |  |
| Lenght of stay | 41 (58) | 20 (67) | 15 (58) | 12 (67) | No agreement | 41 (60) | 17 (59) | 13 (59) | | 10 (67) | | No agreement | |  |
| Neonatal admission to intensive care unit | 64 (89) | 30 (100) | 23 (88) | 17 (89) | Agree to include | NA | NA | NA | | NA | | NA | |  |
| Neonatal mechanical ventilation (invasive and non invasive) | 61 (85) | 25 (83) | 23 (88) | 14 (82) | Agree to include | NA | NA | NA | | NA | | NA | |  |
| Neonatal oxygen support | 56 (78) | 27 (90) | 19 (73) | 15 (83) | No agreement | 64 (93) | 25 (86) | 18 (78) | | 14 (88) | | No agreement | |  |
| Phototherapy requirement | 30 (43) | 11 (37) | 7 (29) | 6 (25) | Agree to exclude | NA | NA | NA | | NA | | NA | |  |
| Skin-to-skin | 36 (51) | 18 (60) | 8 (35) | 16 (84) | No agreement | 32 (47) | 17 (59) | 5 (25) | | 14 (93) | | No agreement* | |  |
| Transfer to a higherlevel health facility | 53 (76) | 24 (80) | 17 (65) | 17 (89) | No agreement | 59 (85) | 23 (79) | 19 (83) | | 14 (88) | | No agreement | |  |
|  |  |  |  |  |  |  |  |  | |  | |  | |  |
| Note: With variations depending on the outcome, on average, 7·7% of the participants in Round 1 and 0·55% in Round 2 abstained from voting because they perceived themselves as unable to rate the outcome due to a lack of expertise· | | | | | | |  | |  | |  | |  |  |
| * These outcomes were deemed borderline if, in the second survey, ≥90% of participants in at least one stakeholder panel rated the outcome as critically important, did not have substantial overlap with included outcomes, were included within domains with substantial gaps, and were relevant to smaller panels· | | | | | | | | | | | | | |  |

1. Dodd S, Clarke M, Becker L, Mavergames C, Fish R, Williamson PR. A taxonomy has been developed for outcomes in medical research to help improve knowledge discovery. J Clin Epidemiol. 2018 Apr;96:84–92.

2. COMET initiative [Internet]. [cited 2024 Apr 3]. Available from: https://www.comet-initiative.org/Studies

3. COSMIN - Improving the selection of outcome measurement instruments [Internet]. COSMIN. 2017 [cited 2024 Apr 3]. Available from: https://www.cosmin.nl/

4. ICD-11 for mortality and morbidity statistics [Internet]. [cited 2023 Jun 8]. Available from: https://icd.who.int/browse11/l-m/en
